# The Botanical, Chemical and Ethnobotanical Diversity of Southern African Lamiaceae

**DOI:** 10.3390/molecules26123712

**Published:** 2021-06-18

**Authors:** Ryan D. Rattray, Ben-Erik Van Wyk

**Affiliations:** Department of Botany and Plant Biotechnology, University of Johannesburg, Auckland Park, P.O. Box 524, Johannesburg 2006, South Africa; ryanr@uj.ac.za

**Keywords:** Lamiaceae, diversity, southern Africa, volatile oils, essential oils, phytochemistry, traditional medicines, useful plants, medicinal plants, food plants

## Abstract

The Lamiaceae is undoubtedly an important plant family, having a rich history of use that spans the globe with many species being used in folk medicine and modern industries alike. Their ability to produce aromatic volatile oils has made them valuable sources of materials in the cosmetic, culinary, and pharmaceutical industries. A thorough account of the taxonomic diversity, chemistry and ethnobotany is lacking for southern African Lamiaceae, which feature some of the region’s most notable medicinal and edible plant species. We provide a comprehensive insight into the Lamiaceae flora of southern Africa, comprising 297 species in 42 genera, 105 of which are endemic to the subcontinent. We further explore the medicinal and traditional uses, where all genera with documented uses are covered for the region. A broad review of the chemistry of southern African Lamiaceae is presented, noting that only 101 species (34%) have been investigated chemically (either their volatile oils or phytochemical characterization of secondary metabolites), thus presenting many and varied opportunities for further studies. The main aim of our study was therefore to present an up-to-date account of the botany, chemistry and traditional uses of the family in southern Africa, and to identify obvious knowledge gaps.

## 1. Introduction

The Lamiaceae Martinov (formerly Labiate) are a commercially important, cosmopolitan family of flowering plants comprising ca. 250 genera and 7825 species [1]. The largest and best-known genera include *Salvia* L. (900 spp.), *Scutellaria* L. (360 spp.), *Stachys* L. (300 spp.), *Plectranthus* L’Hér. (300 spp.), *Hyptis* Jacq. (280 spp.), *Teucrium* L. (250 spp.), *Vitex* L. (250 spp.), *Thymus* L. (220 spp.) and *Nepeta* L. (200 spp.). Many species are highly aromatic and possess complex mixtures of bioactive secondary metabolites which contribute to their global importance as sources of medicinal and culinary herbs. Herbal teas that are rich in aromatic and phenolic compounds, such as chamomile (*Matricaria chamomilla* L.), rooibos tea (*Aspalathus linearis* (Burm.f.) Dahlg.), maté (*Ilex paraguariensis* A.St.-Hil.) and green tea (*Camellia sinensis* (L.) Kuntze) have become very popular [2,3] and it seems worthwhile to systematically explore the commercial potential of poorly known herbal teas. 

A recent global review of ca. 900 commercially important medicinal plant species [2] included 44 members of the Lamiaceae (ca. 5% of the total). Similarly, a review of major food plants [3] listed 817 species, of which 43 (ca. 5%) belongs to the Lamiaceae. The contribution of Lamiaceae species to the culinary herbs and spices of the world is even more prominent with 114 species out of 701 listed (ca. 16%) [4]. The Lamiaceae also features prominently in a family-level analysis of medicinal plants used in Traditional African medicine [5]. Due to their popular use, members of this family play a pivotal role in many industries, including cosmetics, flavoring, fragrance, perfumery, pesticides and pharmaceutical development. On account of their economic importance and wide-spread use, many species have been introduced and cultivated in various countries across the world [6]. 

**Figure 1:**
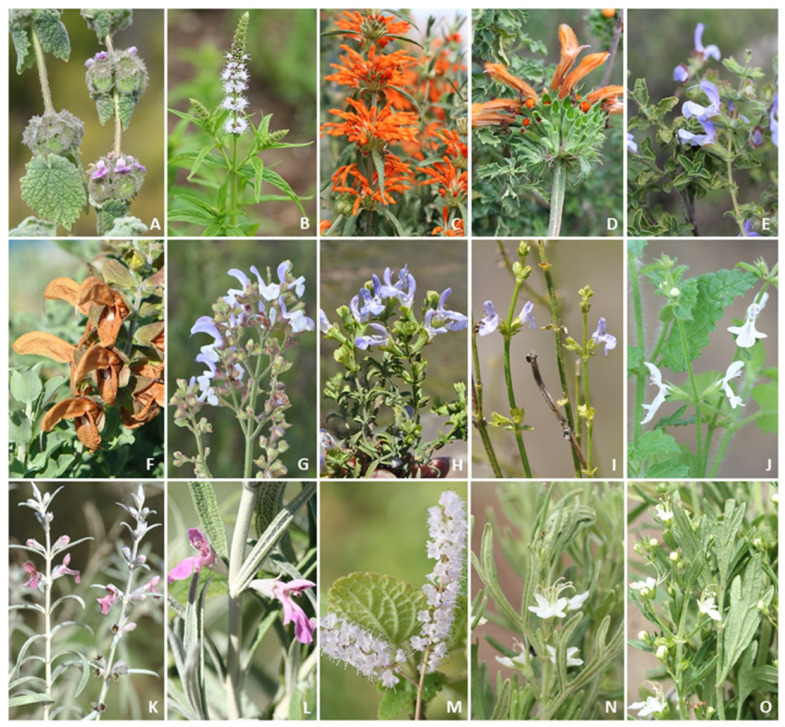
Southern African medicinal plants of the Lamiaceae that are commonly used in traditional medicine: (**A**) *Pseudodictamnus africanus*; (**B**) *Mentha longifolia*; (**C**) *Leonotis leonurus*; (**D**) *L. ocymifolia*; (**E**) *Salvia africana*; (**F**) *S. aurea*; (**G**) *S. chamealaeagnea*; (**H**) *S. dentata*; (**I**) *S. stenophylla*; (**J**) *Stachys aethiopica*; (**K**) *S. linearis*; (**L**) *S. rugosa*; (**M**) *Tetradenia riparia*; (**N**) *Teucrium africanum* and (**O**) *T. trifidum*. All photographs taken by B.-E. Van Wyk.

Southern Africa is not only known for its high levels of plant diversity (20,401 species) and plant endemism (67%) [7] but also for its cultural diversity, resulting in a rich ethnobotanical heritage. Many important aromatic, medicinal and ornamental plants have been used for centuries in traditional medicines by indigenous people across the globe [8]. A bibliography of plants used in traditional medicine in southern Africa [Arnold et al. (2002)] included 215 families, equating to 3689 taxa (comprising species, subspecies and varieties) and represents ca. 15% of the regional flora [9]. Some southern African representatives of the Lamiaceae are well-documented as traditional medicines, including *Leonotis leonurus* (L.) R. Br. and *Salvia africana* L., as well as food and beverage plants (*Coleus esculentus* (N.E. Br.) G. Taylor, *C. rotundifolius* (Poir.) A. Chev. and Perrot and *Mentha aquatica* L.) [10,11,12,13,14]. In a review of 150 important medicinal plants of South Africa (Van Wyk et al. (2009) [15]), 15 species of Lamiaceae that are commonly used in traditional medicine were included (Figure 1). 

**Figure 2:**
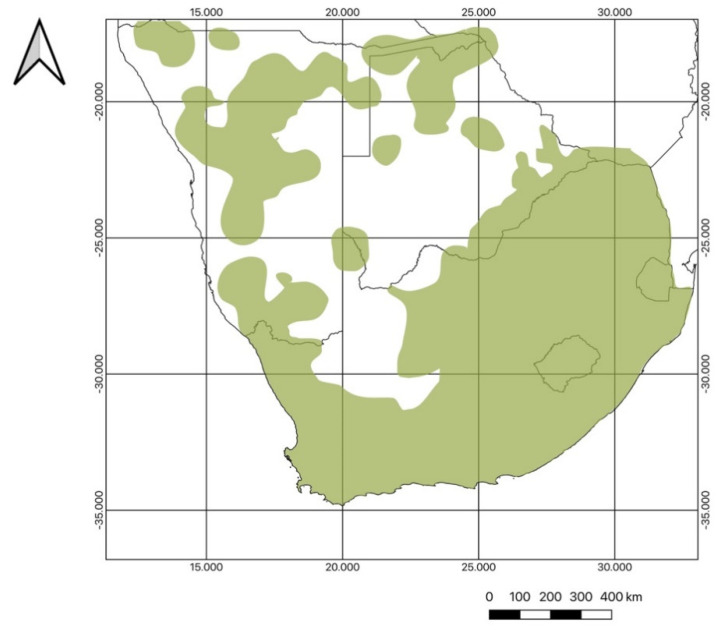
The geographical distribution of the Lamiaceae in southern Africa.

In this detailed review, we provide a broad overview of the botanical diversity, reported volatile and other chemical compounds and traditional uses of the Lamiaceae in southern Africa. The geographical area covered is the Flora of Southern Africa region (Figure 2), which includes South Africa, Botswana, Eswatini (Swaziland), Lesotho and Namibia. Our hypothesis was that the southern African Lamiaceae have remained poorly studied as a result of a lack of clarity on generic delimitations and phylogenetic relationships. The review therefore reflects on the current classification system of the family and provides a summary of the current state of knowledge regarding the secondary metabolites that have been reported in the literature, as well as traditional uses of southern African species (data from recent ethnobotanical surveys are included). The aim was to gain a more profound understanding of the patterns of chemical variation and ethnobotanical diversity, and to identify obvious knowledge gaps that require further study. 

## 2. Botanical Diversity

Members of the Lamiaceae family are generally recognized by a combination of traits which include opposite leaves, bilaterally symmetric flowers with four stamens, and ovaries consisting of two fused carpels, each divided into two one-seeded chambers [16].

**Figure 3:**
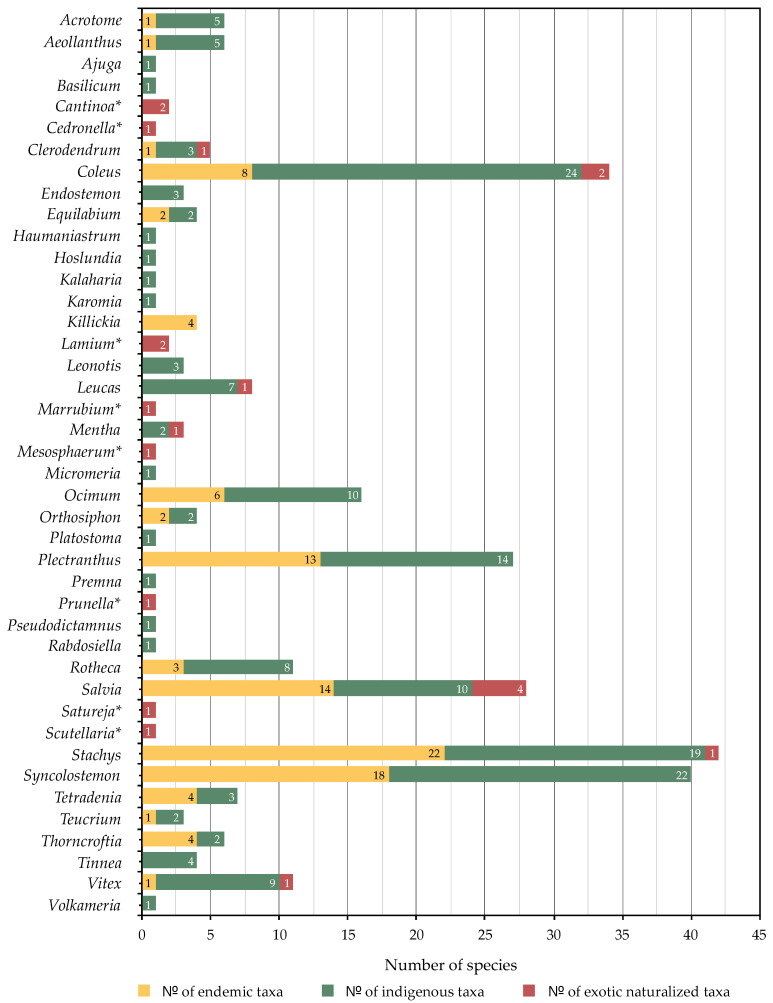
The genera and number of species (endemic, indigenous and naturalized) within southern African Lamiaceae.

Southern African Lamiaceae comprises of 42 genera and 297 species (excluding subspecies and varieties; a full list of the 329 taxa can be found in Table S1) of which 105 species are endemic to the Flora of Southern Africa region (Figure 2), 171 are indigenous to the region and 21 are naturalized exotics (Figure 3). The five largest genera are *Stachys* (41 species), *Syncolostemon* E. Mey. ex Benth. (40 species), *Coleus* Lour. (34 species), *Salvia* (28 species) and *Plectranthus* (27 species). The smallest genera, comprising only a single species each, are: *Ajuga* L., *Basilicum* Moench, *Cedronella* Moench*, *Haumaniastrum* P.A. Duvign. and Plancke, *Hoslundia* Vahl, *Kalaharia* Baill., *Karomia* Dop, *Lamium* L., *Marrubium* L.*, *Mesosphaerum* P. Browne, *Platostoma* P. Beauv., *Premna* L., *Prunella* L.*, *Pseudodictamnus africanus* (L.) Salmaki and Siadati, *Rabdosiella* Codd, *Satureja* L.*, Scutellaria*, and *Volkameria* P. Browne (naturalized exotics are indicated with an asterisk).

The last taxonomic account of southern African Lamiaceae was by Codd in 1985 and included 232 species within 37 genera [17]. Since then, many studies have produced nomenclatural changes, resulting in the movement of one genus to another, or members from one family moved to another, based on molecular data. Several studies illustrate this, including the renaming of many *Plectranthus* species to *Coleus* [18], *Becium* Lindl. to *Ocimum* L. [19], *Hemizygia* (Benth.) Briq. to *Syncolostemon* [20], and members of other families (such as Verbenaceae J. St.-Hil.) being moved to Lamiaceae [21,22], thus making it difficult to find an up-to-date account of Lamiaceae species for the region.

Similarly, the subfamilial and tribal classification of indigenous southern African Lamiaceae genera has only recently reached some stability because of an extensive molecular phylogenetic study [19]. The new classification system of 10 subfamilies provides, for the first time, a framework for future comparative studies to evaluate the chemotaxonomic value of secondary metabolites. The phylogenetic affinities of the indigenous southern African genera are shown in the following summary:

Subfamily 1. Prostantheroideae Luerssen. No southern African genera.
Subfamily 2. Symphorematoideae Briquet. No southern African genera.
Subfamily 3. Viticoideae Briquet. *Vitex* (ca. 250 spp., 10 in southern Africa).
Subfamily 4. Nepetoideae (Dumortier) Luerssen. This is the largest subfamily and contains ca. 118 genera and ca, 3400 species [22]. Rosmarinic acid is a potential synapomorphy for the subfamily. The tribe Mentheae Dumort. includes the genera *Killickia* Bräuchler, Heubl and Doroszenko*, Mentha* L.*, Micromeria* Benth.*, Salvia,* while the tribe *Ocimeae* Dumort. has two subtribes: *Ociminae* J.A. Schmidt, with the genera *Basilicum, Endostemon* N.E. Br*., Haumaniastrum, Hoslundia, Ocimum, Orthosiphon* Benth., *Platostoma* and *Syncolostemon*; subtribe Plectranthinae Endl., with the genera *Aeollanthus* C.Mart. ex Spreng*., Coleus, Equilabium* Mwany., A.J. Paton and Culham*, Plectranthus, Rabdosiella, Tetradenia* Benth. and *Thorncroftia* N.E. Br.
Subfamily 5. Premnoideae B. Li, R. G. Olmstead and P. D. Cantino. *Premna* (50–200 spp., only 1 in southern Africa).
Subfamily 6. Ajugoideae Kosteletzky. Ajuga, Clerodendrum L., Kalaharia, Karomia, Rotheca Raf., Teucrium and Volkameria.
Subfamily 7. Peronematoideae B. Li, R. G. Olmstead and P. D. Cantino. No southern African genera.
Subfamily 8. Scutellarioideae (Dumortier) Caruel. *Tinnea* Kotschy ex Hook. f. (19 species, all endemic to Africa, 4 in southern Africa).
Subfamily 9. Cymarioideae B. Li, R. G. Olmstead and P. D. Cantino. No southern African genera.
Subfamily 10. Lamioideae Harley. This is the largest subfamily in the Old World and is second only to Nepetoideae in terms of the numbers of taxa, with more than 60 genera and ca. 1200 species. It has been suggested [19] that allenic components in the seed oil may be a synapomorphy for the subfamily. Of the 10 tribes recognized [19], only three are represented in southern Africa: Stachydeae with *Stachys,* Marrubieae with *Pseudodictamnus* Fabr. and Leucadeae with *Acrotome* Benth. ex Endl*., Harmsiella* Briq.*, Leonotis* (Pers.) R.Br. and *Leucas* R.Br.

## 3. Chemical Compounds

Several groups and sub-groups of variable circumscriptions exist in the Lamiaceae and as a result, the phytochemistry has been deemed complex by many researchers. The general idea is that there are two major groups within the family that produce secondary metabolites: those that produce volatile terpenoids (essential oil) (subfamily Nepetoideae), and those that produce non-volatile polar compounds and that are poor essential oil producers (subfamily Lamioideae) [23]. This generalization is no longer accurate, partly as a result of realignments of genera and partly because several members of the Lamioideae also produce essential oil. The essential oil producers are said to be highly aromatic and reported to possess diverse phytochemical profiles and as a result, exhibit a wide range of biological activities [24]. Major bioactive constituents such as volatile oil compounds (mostly mono- and sesquiterpenoids), flavonoids and hydroxycinnamic acids have been found in the most common Lamiaceae species which include those such as lemon balm, oregano, peppermint, rosemary, sage and thyme [25]. Some species are chemically highly variable and include several distinct chemotypes, producing varying amounts of major constituents within their volatile oils. Studies on Tunisian *Mentha* species revealed two types, one being pulegone dominated and the other menthone dominated; a study in Iran demonstrated piperitenone- and β-caryophyllene-dominated chemotypes. The essential oil composition of the common sage (*Salvia officinalis* L.) has been reported to vary from the “standard” α-thujone > camphor > 1,8-cineole chemotype, while studies on rosemary (*Salvia rosmarinus* Spenn.) from Saudi Arabia presented three chemotypes, a pattern which has similarly been reported from Algeria, Europe and India [26,27,28]. 

Non-volatile secondary metabolites, especially metabolites from polar fractions, can vary greatly at the species level. Generally, there are six main classes of secondary metabolites found in the Lamiaceae, namely: caffeoylquinic and other phenolic acids, flavonoids, iridoids, lignans, non-volatile terpenoids, and phenylethanoid glycosides [23]. One of the earliest accounts of phytochemical investigations of southern African Lamiaceae was in 1964 when marrubiin was isolated from *Leonotis leonurus* [29]. Since then, several compounds have been isolated and their chemical structures determined, primarily diterpenes [30,31,32,33], phenolic compounds [34], pyrones [35,36,37], and glycosides [38,39,40].

**Figure 4:**
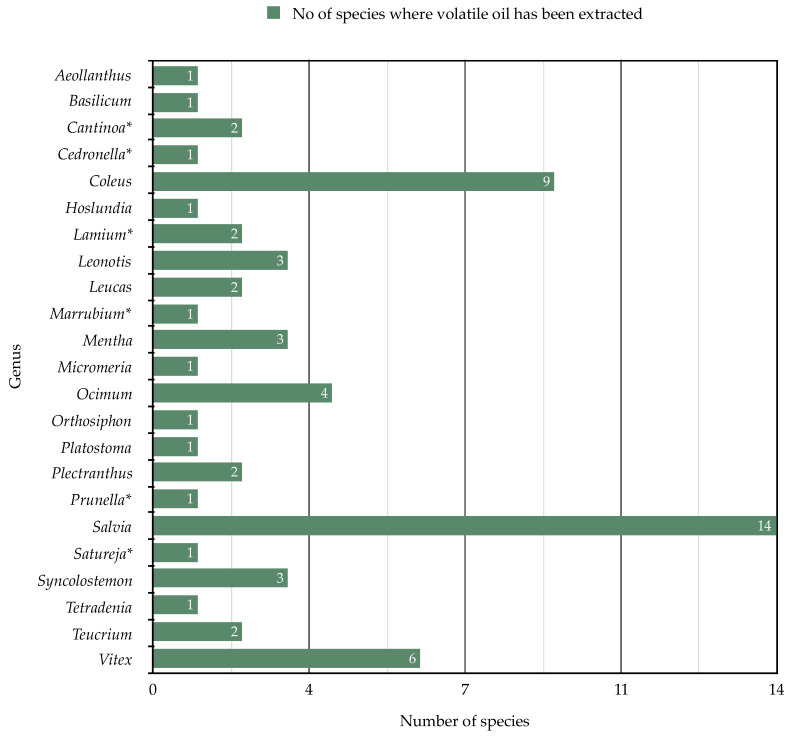
The numbers of species from 23 genera (out of 42) of southern African Lamiaceae that have been studied for their essential oil composition.

**Figure 5:**
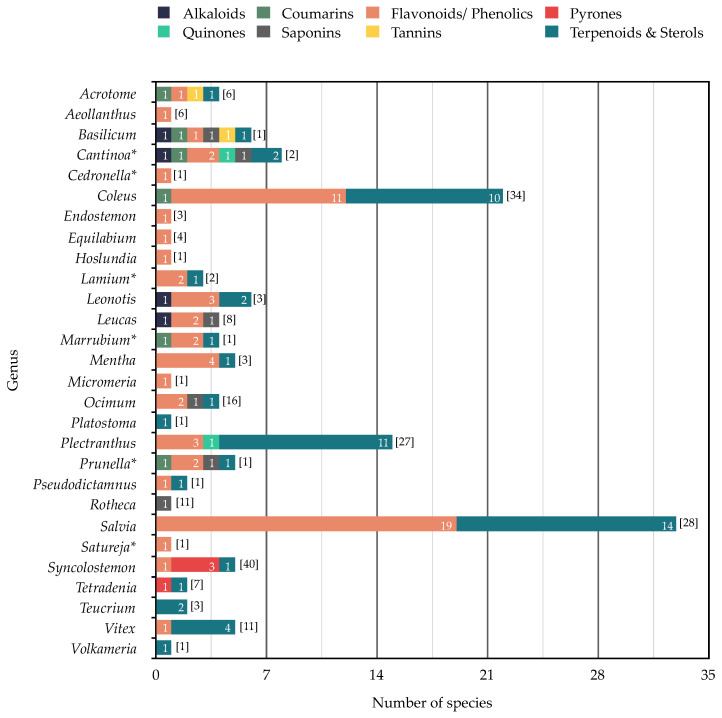
The numbers of species from 28 genera (out of 42) of southern African Lamiaceae that have been subjected to phytochemical studies, and the classes of non-volatile compounds that have been reported (total number of species per genus is shown in square brackets; essential oil studies are shown in Figure 4).

Of the 297 southern African Lamiaceae species, 101 have been chemically studied (i.e., essential oil analyses have been performed or phytochemical extractions and characterization have been made), equating to a total of 34% of species having been chemically profiled. Table 1 provides a summary of genera and species that have been studied and highlights the main compounds that have been recorded. The overall pattern for volatile compounds (Figure 4) shows the relatively small number of genera and species that have hitherto been studied. The same trend is seen in Figure 5 for eight classes of non-volatile secondary metabolites. Below we discuss the different classes of compounds in detail.

### 3.1. Alkaloids

Alkaloids make up a large number of structurally diverse natural products, with more than 27,000 known compounds [2]. They typically exhibit a wide range of biological and mind-altering activities which have led to many becoming important in the pharmaceutical industry [299]. There have been approximately 244 alkaloids isolated and identified from various families of African plants, many of which exhibit antimicrobial, anti-cancer, anti-inflammatory and anti-depressant-like activity [300]. Although alkaloids are relatively rare in the Lamiaceae, they may prove to be of considerable chemosystematic interest. The well-known European wood betony (*Stachys officinalis* (L.) Trev.) accumulates stachydrine and betonicine and it is possible that alkaloids may also occur in one or more of the 41 southern African *Stachys* species (or related genera of the Lamioideae). No information seems to be available, except for phytochemical screening results which suggested that four southern African Lamiaceae tested positive for alkaloids: *Basilicum polystachyon* (L.) Moench, *Cantinoa americana* (Aubl.) Harley and J.F.B. Pastore, *Leonotis leonurus* and *Leucas matrinicensis* (Jacq.) R. Br. [10,38,47,156]. The studies by Eze et al., (2013), Ladan et al., (2014) and Touani et al., (2014) all used the screening method documented in Harborne (1984) [301] and although potentially useful as an indicator, should be followed up by isolation and chemical characterization to confirm the presence of alkaloids. In a review by Nsuala et al. (2015) [10] on *L. leonurus*, the authors state that more definitive research and confirmation are needed. Similarly, it is suggested that more rigorous studies of *B. polystachyon*, *C. americana* and *L. martinicensis* are required.

### 3.2. Coumarins

The simple and versatile structure of the coumarin scaffold has made it a point of interest in many applications such as cosmetics, perfumery and the pharmaceutical industry [302]. Many coumarin compounds are appropriate contenders for modern medications as they possess strong pharmacological activity, lower drug resistance, low toxicity and side-effects, and are highly bioavailable [302]. Coumarins have been isolated and identified in several Lamiaceae species, namely lavender (*Lavandula angustifolia* Mill.), aspic (*L. latifolia* Medik.), European bulge (*Lycopus europaeus* L.), basil (*Ocimum basilicum* L.) and garden sage (*Salvia officinalis*) [302]. Coumarins are reported to be present in six southern African species: *Acrotome inflata* Benth. [34], *Basilicum polystachyon* [47], *Cantinoa ameriacana* [38], *Coleus hadiensis* (Forssk.) A.J. Paton [93], *Marrubium vulgare* L. [164] and *Prunella vulgaris* L. [229].

### 3.3. Phenolics

Phenolics are the second most abundant class of compounds occurring in the data, which is not surprising, as they constitute a large group of secondary metabolites in plants. Furthermore, these compounds are of great interest as nutraceuticals in the food and pharmaceutical industries [303]. Phenolic compounds have been extracted from beverages such as tea and wine, fruits and vegetables, and have undergone an immense number of studies using in vitro methods [304,305,306,307,308]. These studies have identified these compounds as being powerful antioxidant agents, though it has been long debated whether the same in vitro results can be obtained in vivo as the chemistry may alter after being processed by the body [304]. To overcome such issues, phenolic compounds are being loaded into nanocarriers (such as lipid-based nanocarriers, nano-emulsions, nano-scale phospholipids, and nano-structured lipid carriers) all of which are being used to mask their unpleasant flavor in oral administration, providing higher stability and absorption, and better release in gastrointestinal conditions [303]. Moreover, these formulations provide the potential for enhanced solubility, bioavailability and assist in controlled release of the nano-encapsulated phenolic compounds [303]. Apart from their health benefits, compounds such as caffeic acid esters (nepetoidin A and B) appear to be of taxonomic significance within the Lamiaceae and a wider survey may yield useful results. Nepetoidin B was first isolated from the glands of *Coleus caninus* (Roth) Vatke in 1975 [42].

*Coleus amboinicus* Lour. is a popular medicinal plant native to southern Africa, the Arabian Peninsula and India and has been used to treat a wide range of ailments, including digestive problems, skin conditions, respiratory issues, infections and pain [309]. This species is rich in phenolic compounds such as quercetin, vitexin, and coumaric-, caffeic- and cinnamic acids and exhibited very low toxicity with an LC_50_ value of 198.630 µg/mL [60]. The presence of these compounds may contribute to the use of the species as natural and traditional remedies.

Three flavonoids, hoslundin, hoslundal and hoslunddiol, as well as two pyrone-substituted flavonoids—oppositin and 5-*O*-methylhoslundin—have been isolated from Cameroonian *Hoslundia opposita* Vahl [114,116]. In a later study by Salame et al. (2012) [310], two unusual 6-furanoflavones (hoslunfuranine and 5-*O*-methylhoslunfuranine), along with the known methylpyranoflavonic analogues were isolated from the leaves of *H. opposita*. Furthermore, the antileishmanicidal capabilities of select compounds were investigated; two of which exhibited potential in the micromolar range.

In a study by El-Ansari et al. (2009) [137] on *Leonotis leonurus*, ten flavonoid compounds were extracted and identified in the genus for the first time, six of which were flavone glycosides (6-*C*-α-arabinoside-8-*C*-β-glucoside, apigenin 8-*C*-β-glucoside, apigenin-7-*O*-β-glucoside, luteolin 7-*O*-β-glucoside, luteolin 7-*O*-β-glucoside-3′-methyl ether and apigenin 7-*O*-(6″-*O*-*p*-coumaroyl)-β-glucoside), two methylated flavones (6-methoxyluteolin-4′-methyl ether and luteolin 3′-methyl ether) and two flavone aglycones (luteolin and apigenin). Furthermore, the authors investigated the hepatoprotective, anti-inflammatory and cytotoxic activities of methanol and chloroform extracts, observing that these extracts exhibited strong hepatoprotective and anti-inflammatory activity; there was however no cytotoxic activity recorded. Apigenin and luteolin are two examples of phenolic compounds extracted and identified from South African *L. leonurus* [10,140], and Brazilian *Leonotis nepetifolia* (L.) R.Br. Another study in 2015 (Oliveira et al. (2015) [148]), quantified total phenolic and flavonoid content. The extracts from *L. nepetifolia* exhibited a broad-spectrum of antimicrobial activity with strong action against *Shigella flexneri, Enterococcus faecalis*, *Staphylococcus aureus*, *Bacillus subtilis*, *Helicobacter pylori* and *Streptococcus pyogenes* attributed to the relative phenolic content.

Rosmarinic acid, luteolin-7-*O*-glucoside and eriocitrin have been reported as main constituents in the aqueous extract of *Mentha aquatica* [311]. Another study by Benabdallah et al. (2016) [166] determined a high phenolic content in Algerian provenances of *M. aquatica* and noted good antimicrobial activity from the methanol extract. Safaiee et al. (2019) [167] tested the extraction methods for total phenolic compounds in *M. aquatica*. The authors determined that freeze-drying samples, followed by extraction at 60 °C were the most effective at obtaining a maximum yield. In 1973, eight flavonoid compounds were isolated and identified from *Mentha longifolia* (L.) L. namely, luteolin–7–glucoside, luteolin–7–rutinoside, luteolin–7–glucuronide, apigenin–7–glucuronide, acacetin–7–rutinoside, diosmetin–7–rutinoside, hesperetin–7–rutinoside and eriodictyol–7–rutinoside [178]. In a phytochemical study of Moroccan *M. longifolia,* four flavonoids (5,6-dihydroxy-7,8,3′,4′-tetramethoxyflavone, luteolin, luteolin 7-*O*-glucoside, and hesperidin) were isolated, all of which had previously been reported by other studies, while one (5,7,4′-trihydroxy-6,2′,3′-trimethoxyflavone) was reported for the first time from this source [180].

The first phytochemical profiling of non-volatile metabolites in *Micromeria biflora* (Buch.-Ham. ex D. Don) Benth. from Kenya, produced nine flavonoids and caffeic acid oligomers, among which was a newly discovered flavone glycoside, (2″-caffeoyl-luteolin 7-*O*-β-d-glucuronide) [191].

Seventeen of the indigenous southern African *Salvia* species have been investigated. *Salvia aurea* L. was assayed for total phenolic content and the influence that seasonal variation may have [243]. Grzeszczuk, Salachna and Meller (2018) [247] investigated the response of *S. coccinea* Buc’hoz ex Etl. to two concentrations of salicylic acid and increasing concentrations of sodium chloride. They noted that the application of salicylic acid relieved the effects of increased sodium chloride concentrations and subsequently resulted in increased number of branches, higher fresh herbal weight as well as higher contents of total chlorophyll, carotenoids, polyphenols and increased antioxidant activity. In a study of exudate flavonoids in some *Salvia* species, it was noted that *S. stenophylla* Burch. ex Benth. accumulated apigenin, apigenin-7-methyl ether, scutellarein-7,4′-dimethyl ether, luteolin, and 6-hydroxyluteolin-6,7-dimethyl ether. The major constituent of the leaf exudate was not identified [261]. In a study by Kamatou, Viljoen and Steenkamp (2009) [240], sixteen South African *Salvia* species were investigated for their phenolic content. The authors noted that compounds such as betulafolientriol oxide and rosmarinic acid were detected in all the species studied. Furthermore, carnosol, carnosic acid, oleanolic acid/ursolic acid, and rosmarinic acid were abundant in many species [240].

Several glucosides, including lamiol, lamalbid, shanzhiside methyl ester, laminoside and trace amounts of 5-deoxylaminol, sesamoside and barlerine were isolated from *Lamium amplexicaule* L. by Alipieva et al. (2007) [121]. The flavonoid glycoside rutin was detected in *Coleus neochilus* (Schltr.) Codd and *C. madagascariensis* (Pers.) A.Chev. [39]. Flavonoid glycosides and aglycones have been isolated and identified in *Leonotis leonurus* [10], *Lamium galeobdolon* (L.) L. [40], *Mentha aquatica* [175], *M. longifolia* [178] and *Marrubium vulgare* [164] while they have only been detected in *Leucas martinicensis* [156]. A rigorous survey of flavonoids in southern African Lamiaceae may possibly reveal taxonomically useful discontinuities.

### 3.4. Pyrones

α-Pyrones have been isolated and identified in the genera *Syncolostemon* (four species) and *Tetradenia* (two species), both tribe Ocimeae. *Syncolostemon argenteus* N.E. Br. yielded six α-pyrones namely, synargentolides A-E [271], while *S. densiflorus* Benth. gave syndenolide [37,272] and *S. rotundifolius* had synrotolide as main compound [231].

Boronolide has been extracted from *Tetradenia barberae* (N.E. Br.) Codd [231,275] and in *T. riparia* (Hochst.) Codd, umuravumbolide (5,6-dihydro-6-(3-acetoxy-1-heptenyl)-2-pyrone) and desacetylumuravumbolide was isolated for the first time in 1979, and the absolute configuration elucidated in 1995 [35,281]. Natural products belonging to the 6-substituted 5,6-dihydro-α-pyrone family display antifungal, antimicrobial and phytotoxic activities and cytotoxicity against human tumor cells [312]. Comparative studies of geographically representative samples may lead to a better understanding of the chemical variation in *Syncolostemon* and *Tetradenia* species.

### 3.5. Terpenoids and Steroids

Terpenoids, more specifically diterpenoids, are abundant secondary metabolites present in Lamiaceae, as indicated by a large volume of data. Many species contain labdane diterpenoids, a multitude of which demonstrate a broad spectrum of biological activities, including anti-inflammatory, antimicrobial, antiviral, cytotoxic, antioxidant, antihypertensive, and hepatoprotective activities [313,314]. Apart from their functions as important biomolecules, labdane diterpenoids have potential as biomarkers for chemotaxonomic studies and chemical fingerprinting. However, the lack of chromatographic data and reference compounds limits their use in such useful applications [314]. In a review by Hussein (2018) [210], a thorough account of the diterpenoids isolated from southern African species, especially from the genera *Coleus*, *Leonotis*, *Plectranthus*, *Salvia*, and *Tetradenia,* is presented.

In the genus *Coleus*, terpenoids have been extracted from *C. amboinicus*, *C. caninus*, *C. comosus* Hochst. ex Gürke*, C. grandidentatus* (Gürke) A.J. Paton, *C. hereroensis* (Engl.) A.J.Paton, *C. madagascariensis* and *C. porcatus* (van Jaarsv. and P.J.D.Winter) A.J. Paton. Compounds such as coleon U and V, royleanone and horminone were identified from *C. grandidentatus* with coleon U exhibiting potent cytotoxicity against human cancer cell lines and has been deemed a promising anticancer compound needing further investigation [210]. *Coleus comosus* has been well studied with twenty compounds identified, eleven of which are neoclerodanes, seven labdanes and several abietanes [210]. Two abietane diterpenoids were isolated for the first time from *C. madagascariensis* (7β-acetoxy-6β-hydroxyroyleanone and 7β,6β-dihydroxyroyleanone), along with rosmarinic acid and coleon U quinone, all of which exhibited inhibitory activity on α-glucosidase with IC_50_ values ranging from 33 to 275 µM [102]. Furthermore, the abietane diterpenoids exhibited potent antibacterial activities against *Staphylococcus aureus* and *Enterococcus faecalis* [102].

In a study by Achenbach et al., (1992) [113] four abietane-type esters were isolated from the root bark of *Hoslundia opposita*, one of which (identified as 3-*O*-benzoylhosloppone) inhibited the growth of the multidrug resistant strain K_1_ of *Plasmodium falciparum* in vitro with an IC_50_ value of 0.4 μg/mL, thus confirming its use as a traditional African remedy for the treatment of malaria [315].

*Leonotis leonurus* and *L. nepetifolia* are well studied with 21 compounds identified in *L. leonurus* and 30 from *L. nepetifolia*. Marrubiin has been isolated from *L. leonurus* but apparently does not occur in the other two species. Furthermore, the compounds dubiin, leonotin, leonotinin and nepetaefolin occur in *L. nepetifolia* and *L. ocymifolia* (Burm.f.) Iwarsson but has not been observed in *L. leonurus*. Compound ‘X’ however, has been identified in both *L. leonurus* and *O. ocymifolia* [138,153].

Various classes of terpenoids have been identified from the genus *Plectranthus*. Seven tetracyclic phyllocladane-type terpenoids were characterized from *P. ambiguus* (Bolus) Codd, *P. fruticosus* L’Hér. yielded four labdanes and 10 kauranes, some of which exhibited moderate antimicrobial activity [210]. *Plectranthus ecklonii* Benth. and *P. strigosus* Benth. ex E. Mey. both share parviflorone D and F, both of which showed potent antibacterial activity against *Listeria monocytogenes* and *Mycobacterium tuberculosis* while parviflorone D exhibited apoptotic inducing activity in leukemia cells [210]. Other interesting terpenoids isolated from southern African *Plectranthus* include kauranes *ent*-16-kauren-19-ol and *ent*-16-kauren-19oic acid, which have shown antiherpetic properties [210].

Eight *Salvia* species have had terpenoid compounds identified. *Salvia aurea* and *S. chamelaegnea* Berg. have been documented to contain carnosol, whereas *S. chamelaeagnea* and *S. verbenaca* L. contain ursolic acid. Four nerocladanes have been identified in *S. reflexa* Hornem.

Only one species of *Tetradenia* has been investigated for terpenoids in southern Africa, namely *T. riparia.* It was shown to contain several terpenoids, one identified as ibozol [210]. *Tetradenia* is an African-endemic genus that should be further investigated.

### 3.6. Volatile Oils

Volatile oils have been extracted (mainly through steam distillation) from 63 species in 23 genera of southern African Lamiaceae. Twelve of the 23 genera have had all members investigated (mainly those with a single or a few species) and the remaining eleven genera have had oils from some members studied. Furthermore, *Teucrium* and *Vitex* are well-studied with 67% and 60% of their species covered, compared to *Salvia* with only 50% of its members studied.

**Figure 6:**
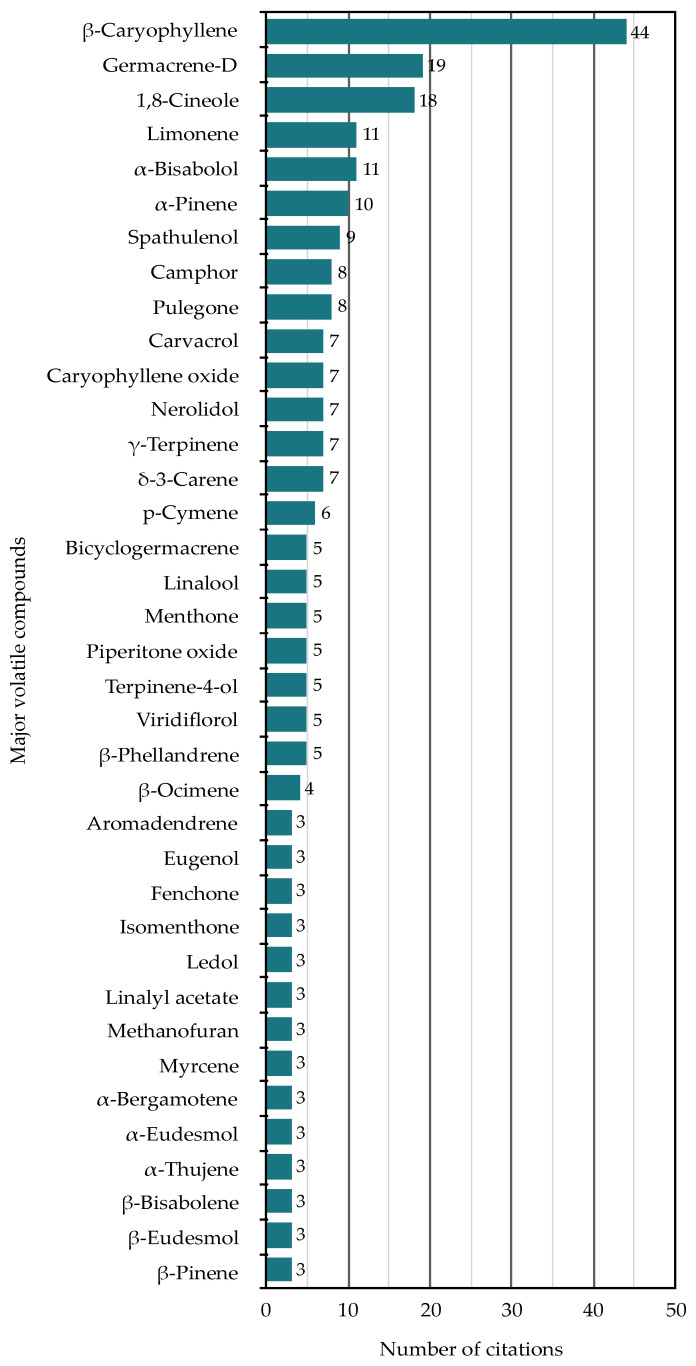
Summary of the frequency of citation of major essential oil compounds (i.e., those representing more than 10% of the composition of the oil) reported from the genera of southern African Lamiaceae. Only compounds with three or more citations are shown.

To reduce the complexity of the data for comparative purposes, only major compounds were listed in Table 1. Major compounds were defined as any essential oil constituent present at a level of at least 10% of the total oil composition. A total of 133 major compounds were thus identified across the 63 species. The frequency of citation of 97 of the 133 compounds (all those with three or more citations) is shown in Figure 6. β-Caryophyllene is by far the most common major compound, followed at some distance by germacrene-D, 1,8-cineole, limonene, α-pinene and α-bisabolol.

It is no surprise that β-caryophyllene is the most common major compound in the essential oil of southern African Lamiaceae as it widely distributed throughout the Plant Kingdom. It contributes to the unique aromas of essential oils and plays a pivotal role in the evolution and survival of higher plants. Furthermore, studies have provided evidence that support β-caryophyllene as a potential therapeutic tool based on the protective roles it exhibits on animal cells [316]. Moreover, experimental results have noted the ability of this molecule to reduce effects of chronic pathologies characterized by inflammation and oxidative stress, especially metabolic and neurological diseases [316]. β-Caryophyllene has exhibited beneficial effects on diabetes, cardiovascular diseases, obesity, some liver diseases, pain and other nervous system disorders [316].

Given the common occurrence of the mono- and sesquiterpenoids in several unrelated families and genera, it is not surprising to find that there are no obvious chemosystematic patterns and that essential oil is apparently produced by most if not all of the subfamilies and tribes. The presence of distinct chemotypes in genera such as *Mentha* and *Salvia* increases the complexity. Comparative studies of geographically representative samples may help to unravel patterns of diversity not previously considered, when relationships were not as well understood as they currently are.

Germacrene-D was reported as a major compound in thirteen of the sixteen genera, followed by β-caryophyllene (9/16) and spathulenol (6/16). Several genera with a single representative studied included *Aeollanthus* (*A. parvifolius* Benth.) with α-muurolol as the major compound; *Basilicum* (*B. polystachyon*) with epiglobulol and yanglene; *Cedronella* (*C. canariensis* Moench) with β-pinene, pinocarvone and *p*-allyl anisole; *Hoslundia* (*H. opposita*) had a single study reporting eugenol as major compound; *Marrubium* (*M. vulgare*) γ-eudesmol and β-caryophyllene; *Mircomeria* (*M. biflora*) exhibiting geranial, neral, germacrene-D and linalool; *Orthosiphon* (*O. thymiflorus* (Roth) Sleesen) has 2-isopropyl-5-methyl-9-methylene-bicyclo-1-decene(4.4.0); *Platostoma* (*P. rotundifolium* (Briq.) A.J.Paton) was reported have germacrene-D, β-caryophyllene and spathulenol; *Prunella* (*P. vulgaris*) germacrene-D and aromadendrene; and *Tetradenia* (*T. riparia*) having fenchone as the most reported major compound.

Studies on both species of *Cantinoa* (*C. americana* and *C. mutabilis* (Rich.) Harley and J.F.B. Pastore) documented β-caryophyllene in both species. However, several studies of *C. mutabilis* reported different major compounds, namely germacrene-D, 1,8-cineole, limonene, spathulenol, and camphor. *Coleus* had eight of its members investigated with *C. caninus*, *C. comosus*, and *C. madagascariensis* documented as having β-caryophyllene as one of their major compounds. Carvacrol and caryophyllene were frequently reported compounds in *C. amboinicus*, and camphor in *C. grandidentatus*. *Lamium amplexicaule* was documented to have camphor and germacrene-D as the major compounds.

For *Leonotis*, germacrene-D and β-caryophyllene were frequently reported for all species within the genus. *Leonotis leonurus* was also recorded to have limonene and α-pinene as additional major compounds. In *Mentha*, major compounds were pulegone and menthone, with *M. aquatica* being the only member containing β-caryophyllene as a major compound. Both *M. aquatica* and *M. longifolia* contained 1,8-cineole in a notable proportion. For *Ocimum*, *O. americanum* L. it was reported to have 1,8-cineole as the major compound in several studies, followed by camphor, while *O. gratissimum* L. had germacrene-D and eugenol, as reported in a single study. *Plectranthus*, *sensu stricto* had only two species investigated, with spathulenol and bicyclogermacrene as major compounds, and sabinyl acetate in *P. fruticosus*.

β-Caryophyllene was noted as the most cited major compound in *Salvia*, recorded in seven of the eighteen species studied. For three closely related *Salvia* species (*S. repens* Burch. ex Benth., *S. runcinata* L.f. and *S. stenophylla*), α-bisabolol was recorded frequently, indicating at least some chemosystematic value. Other major compounds documented for *Salvia* included 1,8-cineole, limonene, and α-pinene. One study recorded α-cubebene and β-cubebene as major compounds in both *Teucrium africanum* Thunb. and *T. sessiliflorum* Benth*. Vitex* was shown to have three species exhibiting 1,8-cineole as one of their major compounds.

In order to make progress in chemosystematic evaluations of essential oil compounds, multiple samples will be essential and multivariate statistical approaches are most likely needed to detect less obvious patterns. It may also be useful to explore the biosynthetic relationships between the individual compounds to ascertain the possible presence or absence of particular biosynthetic pathways that can be used to support or disprove relationships.

## 4. Traditional Uses

Throughout history, there have been numerous accounts of Lamiaceae species used in traditional (folk) medicines and as functional foods. This is likely due to the preserved knowledge regarding their benefits and effects exhibited by their preventative and curative properties. Many ethnic groups across the globe have at least a single recorded account of Lamiaceae as either a cure for an ailment, or the flavoring and preservation of food. Many species native to Europe have been used as culinary herbs and spices, including rosemary, sage and thyme. Their use is not only limited to medicines and food, but also form part of ceremonies, rituals and ‘magic’—such as the use of *Salvia apiana* Jeps. (white sage) by the Native Americans and *Ocimum sanctum* L. (holy basil) in India for prayers and rituals [317,318]. A study in Lebanon documented, for the first time, several genera (*Lavandula* L., *Melissa* L., *Mentha*, *Origanum* L., *Rosmarinus* L., *Salvia*, *Satureja* and *Thymus* L.) used as curatives against gastro-intestinal disorders, hypoglycemia, respiratory disorders, and as cardiotonics and antihypertensives, to name but a few [319]. In China, species such as *Scutellaria baicalensis* Georgi, *Salvia militiorrhiza* Bunge, *Clerodendrum bungei*, and *Leonurus japonicus* Houtt. have well-documented histories in Traditional Chinese Medicine [320,321,322]. It should not be overlooked that the use of local wild resources, in this case the Lamiaceae, must be supported by concepts such as the “ecological economy”, “sustainable development” and “equitable benefit-sharing” [323].

**Figure 7:**
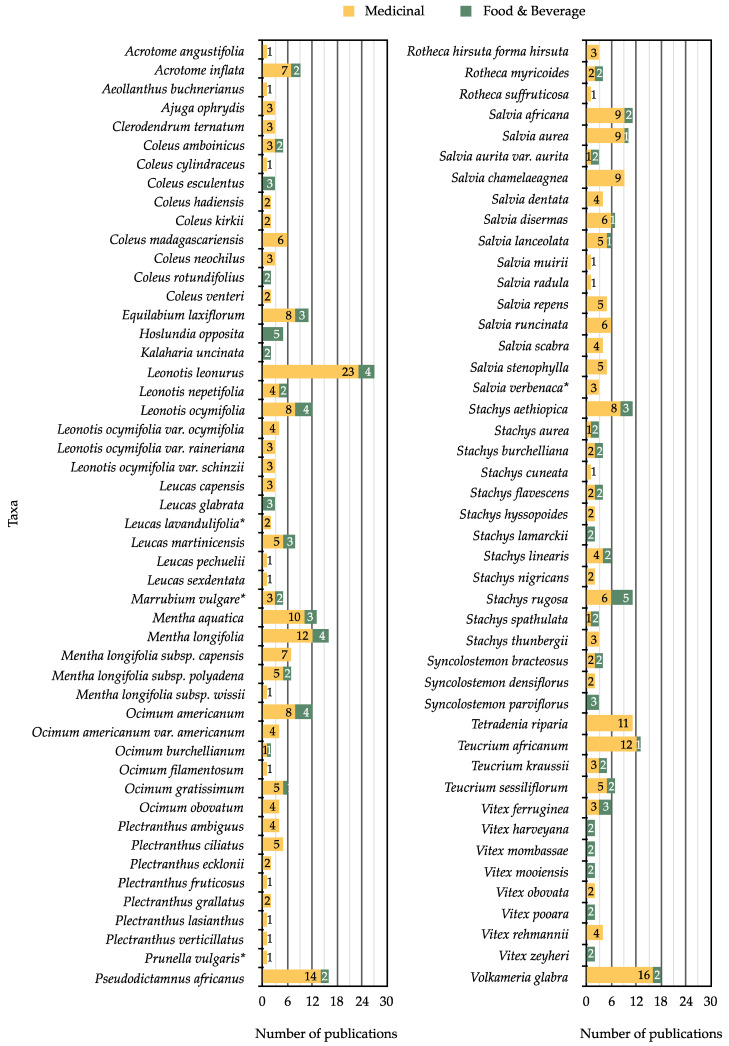
The numbers of publications where medicinal (yellow) and food and beverage (green) uses have been recorded for southern African genera and species of Lamiaceae.

Southern Africa is no different with a wide array of recorded traditional uses, as summarized in Figure 7 (a list of publications used to generate the figure are listed in Supplementary Materials Table S2). Some of these are briefly discussed below.

### 4.1. Medicinal Uses

A total of 83 taxa have documented medicinal uses in southern Africa (Figure 7). The genera with the highest number of records include *Salvia* (68 records), *Leonotis* (45 records), *Mentha* (35 records), *Stachys* (32 records) and *Ocimum* (23 records) (Figure 7). Well-documented species of medicinal value include *Leonotis leonurus* (23 records), *Volkameria glabra* (E.Mey.) Mabb. and Y.W.Yuan (16 records), *Pseudodictamnus africanus* (14 records), *Mentha longifolia* (12 records), *Teucrium africanum* (12 records) and *Tetradenia riparia* (11 records) (Figure 7). Several of these species are noted by Van Wyk et al. (2009) [15] as being part of the 150 best known and most popular herbal medicines in South Africa (Figure 1).

Other species such as *Acrotome angustifolia* G.Taylor is used as a medicinal tea administered to children for upset stomachs whereas *A. inflata* is used in Namibia as a treatment for coughs, fever and breast pain, and in the Eastern Cape of South Africa, it used as a treatment for skin ailments, including chickenpox, wounds, sores, rashes and eczema [17,324,325]. The use of *A. inflata* for topical use is corroborated by Iyambo, Kibuule and Ilonga (2017) [34] who tested methanolic and aqueous extracts of the plant on several bacterial strains, including *Pseudomonas aeruginosa*, *Staphylococcus aureus* and *Bacillus subtilis*. The results produced in the study were comparable with that of penicillin, although the extracts did not show any activity as an antifungal agent against *Candida albicans*. Furthermore, *A. inflata* is used in a multi-ingredient remedy ‘*Sehlare se Seebana*’ by the northern Sotho as a treatment for epilepsy. In a study by Jäger et al. (2005) [326], aqueous and methanol extracts of *A. inflata*, along with five other plant species, were screened using the GABA_A_-benzodiazepine binding assay. Two of the plant species exhibited good dose-dependent activity, although *A. inflata* was not one of them. Aqueous and ethanol extracts were prepared for the six species together and tested, it was noted that the ethanol extract for the mixture was more active than the aqueous one, though the results did not suggest any synergistic effects.

Southern Sotho people administer a traditional medicine made from *Aeollanthus buchnerianus* Briq. as a cold remedy for children, and the use of *Ajuga ophrydis* Burch. ex Benth. to treat reproductive issues such as sterility and infertility is also well documented [327,328]. Compounds isolated from *A. buchnerianus* have been documented to exhibit in vitro antifungal properties against *Cladosporium cucumerinum*, *Aspergillus niger* and *Pythium ultimum*, as well as antimicrobial activity against *B. subtilis*, *S. aureus* and *Streptomyces scabies* with MIC values of 80, 20 and 20 µg/mL respectively [30].

In north-eastern Namibia, *Clerodendrum ternatum* Schinz is documented as a remedy for urinary problems as well as a topical treatment for leg ailments, and in South Africa it has been used by the Bapedi people to treat chronic cough [329,330].

From the genus *Coleus*, *C. amboinicus* has been used medicinally for both humans and livestock [331]. In a study by Sahrial and Solfaine (2019) [332], the authors noted that the ethanolic extract of *C. amboinicus* administered to Wistar rats with cisplatin-induced nephropathy inhibited pathological lesions by regulating the renal expression of TGF-1β in areas containing renal glomeruli and tubules. Furthermore, *C. amboinicus* has shown to exhibit moderate to high antibacterial [66,67,333,334], antioxidant/cytoprotective [333,335,336], and anticancer [336,337] activity.

The Zulu people have used *C. hadiensis* for treating chest ailments such as coughs, *C. kirkii* as a mouthwash for aching teeth caused by neuralgia, and *C. madagascariensis* for respiratory ailments and topical use [338]. *Coleus hadiensis* has been reported to exhibit moderate antimicrobial activity against *Sclerotinia sclerotiorum*, *Candida* species, *B. subtilis* and *Xanthomonas campestris* producing inhibition zones of 16 mm, 15 mm, 8 mm and 10 mm respectively [96]. Another study by Menon, Sasikumar and Latha (2011) [339], in vitro anti-inflammatory and cytotoxic activity of *C. hadiensis* were studied. The authors noted that the plant extract at a concentration of 1 mg/mL exhibited 86.10% BSA denaturation inhibition, 87.49% platelet aggregation inhibition and 87.26% HRBC membrane stabilization, results similar to the standard non-steroidal drug, Dicoflenac. Furthermore, an IC_50_ value of 141.3 µg/mL was recorded for the cytotoxicity against HeLa cells, suggesting that the methanolic extract of *C. hadiensis* has promising therapeutic potential. For *C. madagascariensis* bactericidal activity against *B. subtilis*, *Micrococcus* species, *S. aureus*, and *Yersinia enterocolitica* have been recorded, and also revealed to show modest antioxidant activity [101]. In another study, the acetone extract of *C. madagascariensis* exhibited potent antibacterial activity against Gram-positive bacteria with MIC values ranging from 1.95 to 7.81 µg/mL, and moderate activity against Gram-negative bacteria with MIC values ranging from 0.48 to 3.91 µg/mL [39]. Moreover, potent antioxidant activity was noted, and moderate cytotoxicity was recorded against triple negative human breast carcinoma [39]. *Coleus venteri* (van Jaarsv. and Hankey) A.J.Paton has been documented for treating influenza, blocked nasal passages and high blood pressure in Central Sekhukhuneland, South Africa [340,341]. A study by Maree et al., (2014) [111] investigated the inhibitory activities of two bioactive acetophenones from *C. venteri*, where the authors noted marked inhibitory activities against the transfer of the IncW plasmid R7K in a bacterial plasmid transfer inhibition assay.

*Equilabium laxiflorum* (Benth.) Mwany. and A.J. Paton has been documented to treat a wide range of ailments including troubled eyes, colds, influenza, stomach upset, bleeding gums and fever [338,342,343]. A study by Maharaj et al. (2010) [344] screened the two-minute mosquito repellency of aqueous and organic extracts of South African medicinal plants in a rodent model; *E. laxiflorum* being one of them. The authors noted 55%, 53% and 38% repellency for aqueous, organic and 1:1 aqueous-organic *E. laxiflorum* extracts respectively.

Recorded medicinal uses of *Hoslundia opposita* include the treatment of cystitis, liver disease, stomach ailments, gonorrhea, eye concerns and snakebites [345]. The acute toxicity of the ethanolic extract of *H. opposita* was investigated by Oloyede and Akindele (2020) [346] in Swiss mice (*Mus musculus*). The study concluded that *H. opposita* is devoid of acute toxicosis at the examined doses of 50, 100 and 200 mg/kg over a period of fourteen days. Other studies have investigated the central nervous system depressant activity of *H. opposita*. A study by Olajide, Awe and Makinde (1999) [347] observed that the chloroform extract of *H. opposita* significantly enhanced the phenobarbitone sleeping time in mice and produced a 60% protection against leptazol-induced convulsions. Another study by Risa et al. (2004) [348] noted the ethanolic leaf extract of *H. opposita,* among several other plant extracts, to be the most active against epilepsy and convulsions using the GABA_A_-benzodiazepine receptor assay.

The genus *Leonotis* has many documented medicinal uses. The crushed leaves of *L. leonurus* are used to prepare a decoction which is taken orally twice daily to assist in weight-loss and as an anti-diabetic aid [349,350,351]. Furthermore, it has been documented to treat cancer, ulcers, gout, as well as aches and pains [11]. The pharmacology of *L. leonurus* is well-recorded as reviewed by Mazimba (2015) [140] and Nsuala et al. (2015) [10], though some notable studies include the in vitro anti-HIV activity of several South African medicinal plant extracts, of which *L. leonurus* was recorded to exhibit significant HIV-1 inhibition (33% reduction in HIV-1 p24, *p* < 0.05) [352]. Other studies have observed noteworthy anti-inflammatory and analgesic activity [137], as well as anthelmintic [353,354], hepatoprotective [137] and anti-diabetic activities [355]. Furthermore, the presence of marrubiin in several Lamiaceae species and the pharmacological efficacy of this terpenoid has been well documented. Several studies and reviews illustrated the effect of *L. leonurus* extracts as cardioprotective, antidiabetic, gastroprotective, antispasmodic and analgesic agents [10,356,357,358]. *Leonotis nepetifolia* has been used to treat influenza, chest conditions, insect stings and snake bites, whereas *L. ocymifolia* and its varieties have documented uses to treat high blood pressure, poor blood circulation, rheumatism, diabetes, nerve weakness and snakebites [359,360,361]. A study on Brazilian *L. nepetifolia* by de Oliveira et al. (2019) [362], noted potent antileishmanial activity from leaf and root extracts (IC_50_ values of 32.90 µg/mL and 57.70 µg/mL respectively), while leaf extracts inhibited *Bacillus cereus* (125 µg/mL), and *S. aureus* (100 µg/mL), as well as exhibiting anti-*Candida* activity (IC_50_ values ranging from 10–125 µg/mL). A study by Oyedeji and Afolayan (2005) [129] on the antimicrobial activity of both *L. leonurus* and *L. ocymifolia* from the Eastern Cape, revealed that both species exhibited a broad spectrum of antimicrobial activity against both Gram-positive and Gram-negative microorganisms with MIC values ranging from 0.039–1.25 mg/mL.

*Leucas capensis* (Benth.) Engl. has been used for oral hygiene, headaches, sore eyes, and a treatment for hemorrhoids and chest ailments [17,327,363]. *Leucas lavandulifolia* Sm., *L. martinicensis*, *L. pechuelii* (Kuntze) Baker and *L. sexdentata* Skan have been used to treat conditions such as fever, aches, pains, respiratory ailments, stomach cramps, dizziness, and gastro-intestinal and skin ailments [345,364,365]. *Leucas lavandulifolia* has shown to exhibit significant in vivo antidiabetic, antioxidant and hepatoprotective activities in rats [366,367], while *L. martinicensis* has been reported as having notable antioxidant activity [368] although extracts are relatively safe, prolonged use carry the risk of cardiac toxicities [156].

The naturalized exotic, *Marrubium vulgare*, has been documented to treat respiratory conditions, fever and inflammation [343,369,370]. In reviews by Lodhi et al. (2017) [371] and Yabrir (2019) [372], the authors provide an in-depth view of the pharmacological activity of *M. vulgare* which include analgesic, anti-inflammatory, anti-spasmodic, immunomodulatory, antimicrobial and cytotoxic activities, to mention a few.

*Mentha longifolia* is used as a treatment for coughs, colds, asthma and other respiratory ailments [13]. A tea is prepared from the leaves and said to alleviate colds and flu, as well assist with stomach problems. The warm leaves as used as a compress to treat headaches [373,374]. In a study in 1997, McGaw, Jäger and Van Staden (1997) [375] investigated the ability of 26 South African traditional medicines to inhibit prostaglandin synthesis. Of the species investigated, *M. longifolia* exhibited moderate to higher activity (52–91%) with increasing amount of extract (50–100 µg); the organic extract had the higher potential of the extracts tested. A study from Iraq investigated the antimicrobial activity of *M. longifolia* based on its use as a folk remedy for sore throats and oral irritations. Menthone was isolated and identified as the antibacterial compound which exhibited excellent antimicrobial activity against several clinical pathogens, thus validating the plants use in the treatment for oral concerns [376]. Similarly, *M. longifolia* from Algeria presented comparable results where the organic extracts displayed favorable antimicrobial activity against several pathogens which as attributed to the high phenolic content [377].

In the genus *Ocimum*, the highly aromatic *O. americanum* has been used both cosmetically (as a perfume powder), topically (for burns and wounds) and orally as a medicine for chest complaints such as asthma [329,343,378]. The smoke is said to be inhaled as a remedy to stop nosebleeds [379]. *Ocimum burchellianum* Benth. and *O. gratissimum* have documented uses as medicinal teas, while *O. filamentosum* Forssk. and *O. obovatum* E.Mey. ex Benth. have both been used as a treatment to aid hair growth [17,338,343,361]. The volatile oil of *O. americanum* exhibited excellent antimicrobial activity against several pathogens, including *E. faecalis, Enterococcus faecium, Proteus vulgaris, S. aureus* and *S. epidermis* [380]. In a study by Cavalcanti et al. (2004) [381], both *O. americanum* and *O. gratissimum* exhibited excellent larvicidal activity against *Aedes aegypti* with LC_50_ values of 67 and 60 ppm respectively, thus suggesting their potential use for the control of mosquitos. The antimicrobial activity of *O. obovatum* ethanol extract reported excellent activity against *B. subtilis* with an MIC value of 0.074 mg/mL, indicating its use to treat gastro-intestinal ailments may indeed be effective [382].

*Plectranthus* has several species with documented accounts of medicinal use. *Plectranthus ambiguus* has been used by the Zulu people as a treatment for skin sores, chest complaints, tonsilitis, coughs, fever and eye problems [364]. *Plectranthus ciliatus* E. Mey. has been used as both an analgesic, ophthalmic and a soap-substitute to wash sheep skins; *P. grallatus* Briq. has been used in a similar manner [327,359,383]. Antimicrobial studies conducted on *P. ciliatus* presented overall poor activity on test organisms, although moderate activity was recorded against *S. sclerotiorum* and *B. cereus* [91,96]. *Plectranthus ecklonii* exhibited excellent antimicrobial activity against *Streptococcus sobrinus* and *S. mutans* with MIC values of 4.7 and 5.0 mg/mL respectively [384].

*Rotheca hirsuta* (Hochst.) R. Fern. has documented use as a treatment for intestinal worms, urinary infections and scrofula swellings [328,331,338], while both *R. myricoides* (Hochst.) Steane and Mabb. and *R. suffruticosa* (Gürke) Verdc. are said to be used as treatment for snakebites [338,385]. *Rotheca myricoides* has been reported to exhibit excellent in vitro antimicrobial activity against methicillin-resistant *S. aureus, S. aureus, Escherichia coli, Shigella sonnei, C. albicans* and *Mycobacterium tuberculosis* [386]. Other studies have reported that the freeze-dried extracts of *R. myricoides* possessed potent antihyperglycemic and antidyslipidemic effects in vivo [387], though prolonged treatment with *R. myricoides* extracts in mice have reported to cause reduction in body weight, damage to kidneys and liver, and changes in some hematological and biochemical parameters [388].

*Pseudodictamnus africanus* (=*Ballota africana* (L.) Benth.), known locally as ‘*kattekruie*’, is used as a treatment for stomach and heart problems, and is said to improve blood circulation [11]. Other documented uses include the treatment of fever, measles, colds and flu, asthma, bronchitis, headaches and hysteria [15,389]. *Pseudodictamnus africanus* displayed excellent antimicrobial activity against respiratory pathogens (*Streptococcus pyogens*, *Klebsiella pneumoniae* and *S. aureus*) and as an antifungal agent against *C. albicans*. Furthermore, extracts of the plant has exhibited 80–100% affinity for histamine binding, thus making it an excellent antihistamine [390]. Cock and Van Vuuren (2014) [391] screened thirteen South African plant species for their antimicrobial activity against *K. pneumoniae* and its subsequent cause of inflammation. They found among others, that *P. africanus* displayed *K. pneumoniae* inhibition with MIC values below 1000 µg/mL.

*Salvia africana* has several accounts as a remedy for colds, coughs, menstrual complaints and diarrhea [11,389,392]. This species has been documented to exhibit high antioxidant activity and showed potential as an anticancer agent against breast cancer cells [236]. Furthermore, antimicrobial studies using the essential oil have indicated fair to moderate activity [237,238]. Other *Salvia* species such as *S. aurea*, *S. aurita* L.f., *S. chamelaeagnea*, *S. dentata* Aiton, *S. disermas* L. and *S. lanceolata* Lam. are used to treat respiratory ailments, coughs, influenza, fever, and inflammation [351,359,393,394]. *Salvia repens* and *S. runcinata* are used topically to treat burns, sores and dermatological conditions, whereas *S. scabra* Thunb. is documented as a remedy for pediatric conditions [327,351,395]. An antimicrobial study on the volatile oils of *S. aurea*, *S. aurita*, *S. chamelaegena*, *S. disermas*, *S. lanceolata*, *S. repens* and *S. runcinata* exhibited moderate activity against *E. coli, K. pneumoniae, B. cereus* and *S. aureus* with MIC values ranging from 0.03 to 6.00 mg/mL, while moderate antimycobacterial activity with an MIC value of 0.50 mg/mL was recorded for all oils [244]. Another study by Kamatou, Viljoen and Steenkamp (2009) [240] investigated the antioxidant and anti-inflammatory activities of sixteen South African *Salvia* species. The authors noted that majority of the extracts displayed antioxidant activity (IC_50_ values ranging from 1.6 to 74.5 μg/mL and 11.9 to 69.3 μg/mL by means of the 2,2-diphenyl-1-picrylhydrazyl and 2,2′-azino-bis (3-ethylbenzothiazoline-6-sulfonic acid) scavenging assays), while fifteen of the extracts displayed poor anti-inflammatory activity (all IC_50_ values greater than 100 µg/mL with the exception of *S. radula* which presented an IC_50_ value of 78.8 µg/mL) using the 5-lipoxygenase assay [240].

Though mainly used as a tea, the genus *Stachys* has several medicinal accounts. *Stachys aethiopica* L. is used topically to clean and disinfect wounds, as well as to treat respiratory ailments, colds, bronchitis and influenza [351]. *Stachys aurea* Benth., *S. burchelliana* Launert, *S. flavescens* Benth.*,* and *S. hyssopoides* Burch. ex Benth. are used for chest and respiratory ailments [17,378,393,396]; while *S. linearis* Burch. ex Benth. and *S. rugosa* Aiton are used both as a lactogogue and as a lotion for wounds [343,393].

The leaves of *Tetradenia riparia* are highly aromatic and are used throughout Africa to treat coughs, sore throats, fever, boils, mumps, malaria and dengue fever [268,364,397]. In southern Africa however, strong focus as a topical treatment has been documented due to its wound-healing and dermatological capabilities. The organic and aqueous extracts have indicated moderate to strong activity against bacteria and fungi [398,399,400,401,402]. In other studies, the efficacy of *T. riparia* as a treatment for malaria have been investigated with extracts demonstrating moderate anti-malarial activity against two strains of *Plasmodium falciparum* [403].

The genus *Teucrium* have been used ethnopharmacologically for centuries as treatments for ailments ranging from gastrointestinal disorders, respiratory ailments, inflammation and rheumatism [404], documented in both European and southern African literature. Three southern African-endemic species, *T. africanum*, *T. kraussii* Codd and *T. sessiliflorum* have been recorded as a treatment for stomach ailments, colds and flu, snakebites, prostate problems, sore throats, indigestion and as a general tonic for good health [15,289,351,389]. In ethnoveterinary medicine, *T. africanum* is used against gall sickness in cattle, heartworm and bloating in livestock such as goats and sheep [405]. All three species have been investigated for antimicrobial activity with *T. africanum* showing some activity against *Escherichia coli* (with an MIC value of 0.13 mg/mL), *T. kraussii* against *S. pyogenes* (with an MIC value of 0.8 mg/mL) and *T. sessiliflorum* exhibiting activity against *P. aerginosa* (with an MIC value of 0.5 mg/mL) [289,406].

*Volkameria glabra*, the only representative of this genus, has been used to treat a variety of ailments. The Zulu people use it as a treatment for internal worms and parasites, coughs, fevers, respiratory and circulatory issues, as well as gastro-intestinal and reproductive ailments [338,364]. Other documented uses include the treatment of snakebites and skin irritations, and the cleaning of wounds [343,365,385,407]. Extracts of *V. glabra* have shown to exhibit anti-bacterial, anti-fungal, anti-leishmanial and anti-plasmodial activity [408,409,410,411,412].

The in vitro and in vivo pharmacological activities noted by various studies for many southern African Lamiaceae further corroborate their use as natural remedies for the treatment of a wide range of ailments.

### 4.2. Food Uses

Africa has contributed several crops used as food sources worldwide. In Kunkel (1983) [413] the author accounts for ca. 12,000 plant species considered edible by humans, many of which are native to Africa and include species such as coffee (*Coffea arabica* L.), yams (genus *Dioscorea* Plum. ex L.) and African oil palm (*Elaeis guineensis* Jacq.). In Ethiopia, several Lamiaceae species are used as food sources, including *Leucas calostachys* Oliv. (as a famine food), *Salvia dianthera* Roth ex Roem. and Schult (as a vegetable) and *Mentha aquatica* (as a culinary herb) [414].

#### 4.2.1. Fruits and Vegetables

Forty-five (45) species have documented uses as being edible [415,416,417,418,419,420,421,422,423,424,425] with *H. opposita* (five records), *L. leonurus* (four records), and several *Vitex* species (13 records) having the highest number of citations.

The stem tubers of *Coleus esculentus* and the root tubers of *C. rotundifolius* are excellent sources of nutrients and were probably once major sources of starch but they have largely been replaced by the introduction of high-yielding potatoes and sweet potatoes. *Coleus esculentus* is a highly adaptable species and can grow in almost any climatic zone, provided rainfall is moderate and soil has good drainage. Furthermore, the plant’s proclivity to survive difficult conditions make it an excellent candidate as an alternative crop [415,416,417,418]. The sugary fruits of *Hoslundia opposita*, *Vitex ferruginea* Schumach. and Thonn., *V. harveyana* H. Pearson, *V. mombassae* Vatke and *V. mooiensis* H. Pearson are enjoyed as a snack by local cultural groups. The leaves of *L. leonurus* are sometimes used as a leafy vegetable substitute or spinach [23,418,419]. Other species such as *Mentha aquatica*, *M. longifolia* and *Ocimum americanum* are used as flavorants or condiments with meals [363,418].

#### 4.2.2. Beverages

*Stachys*, *Mentha* and *Salvia* had the most documented uses as beverage plants, with twenty, nine and seven records respectively. Generally, hot infusions are prepared from *Mentha aquatica*, *M. longifolia*, *Ocimum americanum*, *Salvia africana* and several *Stachys* species, as likely alternatives to coffee or tea [14,418,419]. In some instances, *M. longifolia* is used to flavor water, as documented by Hulley (2018) [363]. The traditional use of several species of *Stachys* as teas and tonic in southern Africa and the obvious lack of chemical information on these species represent a significant knowledge gap.

### 4.3. Other Uses

The aromatic characteristic that many Lamiaceae species possess make then useful as insect repellents and deterrents. On some occasions, plants are burnt in huts, to fumigate after an illness and to ward off unwanted insects. The Sotho people have used *Salvia repens*, *S. runcinata* and *S. stenophylla* for this purpose [328]. *Coleus neochilus* and *C. cylindraceus* (Hochst. ex Benth.) A.J. Paton have been used as deterrents for flies, mosquitos, and snakes. They are either planted around the homestead, or crushed leaves are placed inside the house [338,341,359]. The highly aromatic leaves of *Ocimum americanum* and *O. gratissimum* have been used by local people as a perfume powder for the body, and as an insect repellent [359,378].

## 5. Discussion

This review shows that some progress has been made towards unravelling the chemical diversity in southern African Lamiaceae but also revealed that several genera and many species remain to be studied. Our hypothesis that the southern African Lamiaceae have remained scientifically poorly explored can therefore not be rejected. Considerable progress is likely to be made by using modern phytochemical methods, such as liquid chromatography-mass spectrometry and metabolomics. Such an approach will not only serve to rapidly extend the coverage of taxa, but also to re-examine even those species that were apparently well studied using classical methods.

Once a more complete picture of the chemical diversity in medicinal species emerges, it will be rewarding to relate the biological activities of individual compounds to the ailments treated, and to explain the popularity and rationale behind the traditional remedies. Several species are used as teas and tonics (especially in the chemically poorly known southern African species of *Stachys*), suggesting potential as new functional food products. It is likely that individual compounds act in an additive or even synergistic way, which complicates the way in which pharmacological studies can be approached. Given their chemical complexity, it is also likely that different compounds or classes of compounds in the Lamiaceae act on different organ systems, resulting in the maintenance and improvement of health. Unlike pure chemical entities used in most modern pharmaceutical products, some compounds in chemically diverse herbal products may act on the respiratory system, others on the gastro-intestinal system and some perhaps directly on the brain, to generate or improve a sense of well-being. One of the first studies to demonstrate powerful antimicrobial synergy [426] was the combination of camphor and 1,8-cineole as main compounds in *Osmitopsis asteriscoides* (L.) Cass. (Asteraceae), a popular traditional medicine used in Cape Herbal Medicine [389]. It is likely that our understanding of the efficacy of traditional medicines will increase once their chemical composition and associated pharmacological activities become better known. Our review can be used as a starting point for future explorations of the taxonomy, chemosystematics, chemistry and pharmacological activity of southern African Lamiaceae, especially to fill in the obvious gaps in our knowledge of hitherto poorly studied taxa. 

## 6. Materials and Methods

Species data was collected from Codd (1985) [17] and the South African National Biodiversity Institute’s ‘*Plants of southern Africa*’ website (www.newposa.sanbi.org). Scientific names and synonyms were validated through Kew’s ‘*Plants of the World*’ database (www.plantsoftheworldonline.org) as it was found to be the most up-to-date database.

Literature searches were conducted by searching several scientific electronic databases, including GoogleScholar (www.scholar.google.com), EBSCOhost (www.ebsco.com), PubMed (www.pubmed.ncbi.nlm.nih.gov), SciFinder (www.scifinder.cas.org), ScienceDirect (www.sciencedirect.com), Springer (www.springer.com) and Wiley Online Library (www.onlinelibrary.wiley.com). Key words were used to search for literature, and this was conducted in the following manner: (“Species name” AND “synonyms” AND “chem*”), (“Species name” AND “synonyms” AND “med*”) and (“Species name” AND “synonyms” AND “traditional use”). A collection of scientific papers, books, dissertations and theses, and unpublished sources were compiled.

Occurrence data were downloaded from the ‘*Global Biodiversity Information Facility*’ (www.GBIF.org (accessed on 16 May 2021)) GBIF Occurrence Download (https://doi.org/10.15468/dl.b8zqjh) and distribution maps generated in QGIS (QGIS Development Team (2021)). QGIS Geographic Information System. Open-Source Geospatial Foundation Project (http://qgis.osgeo.org).

Data was cleaned, sorted analyzed in Microsoft Excel and the graphs were generated in Apple Numbers.

## 7. Conclusions

Lamiaceae is undoubtedly an important and diverse plant family not only globally, but in southern Africa as well. The phytochemical and aromatic diversity have made members of this family of potential value to the food, beverage, cosmetic and pharmaceutical industries. The popular use of Lamiaceae species as traditional medicines and food sources span across the globe and their uses are supported by scientific research. The largely unstudied diversity of chemical constituents and volatile oils of southern African Lamiaceae offer many potential applications as new functional foods and herbal remedies. Southern Africa holds an abundance of opportunities for further research within this diverse and commercially relevant group, with respectively 66% and 71% of its species yet to be explored chemically and ethnobotanically.

It is likely that our understanding of the efficacy of traditional medicines will increase once their chemical composition and associated pharmacological activities become better known. Our review provides clarity on the current state of knowledge on the taxonomy and chemistry of southern African Lamiaceae and associated key publications and will hopefully serve as a useful framework to guide future phytochemical and ethnopharmacological studies.

## Figures and Tables

**Table 1:** Summary of major essential (volatile) oil compounds and phytochemistry data collected for southern African Lamiaceae species. Only the main constituents for essential oils (compounds representing >10% of total yield) and the main classes of compounds for phytochemical studies are listed.

Genus	Species	Main Compounds in Essential Oil ^	Number of Samples Analyzed	Number of Compounds Reported	Reference	Quality of Study	Other Phytochemical Compounds	Reference	Level of Study
*Acrotome*	*A. inflata*	-	-	-	-	-	Coumarins Flavonoids Tannins	[34]	★
							Terpenoids	[41]	★
*Aeollanthus*	*A. buchnerianus*	-	-	-	-	-	Caffeic acid esters	[42]	★★
							Diterpenoids	[30]	★★★
	*A. parvifolius*	α-Muurolol	1 ^a^	26	[43]	★★	-	-	-
*Basilicum*	*B. polystachyon*	Epiglobulol Ylangene	1 ^a^	64	[44]	★★★	Diterpenoids	[45]	★★★
							Stachyonic acid A	[46]	★★★
							Alkaloids Coumarins Flavonoids Phenols Tannins Triterpenes Sterols Saponins	[47]	★
*Cantinoa* *	*C. americana* *	α-Bergamotene β-Caryophyllene Germacrene-A	1 ^b^	30	[48]	★★	Spicigera Lactone	[49]	★★
		δ-3-Carene β-Caryophyllene	1 ^a^	34	[50]	★★	Labdane diterpenes	[51]	★★★
							Alkaloids	[38]	★
		-	-	-	-	-	Carbohydrates Cardiac glycosides Coumarins Flavonoids	[38]	★
*Cantinoa* * (Cont.)	*C. americana* * (Cont.)	-	-	-	-	-	Quinones Resins Saponins Steroids Terpenoids Vitannins	[38]	★
	*C. mutabilis* *	Bicyclogermacrene β-Caryophyllene Curzerene Germacrene-D	2 ^c^	24	[52]	★★	Triterpenoids	[53]	★★★
		1,8-Cineole Limonene Spathulenol	12 ^#^	105	[54]	★★★			
		Camphor cis-Dihydrocarvone trans-Dihydrocarvone	1 ^a^	31	[55]	★★			
*Cedronella* *	*C. canariensis* *	β-Pinene Pinocarvone *p*-Allyl anisole	2 ^a^ -	27 -	[56] -	★★ -	Terpenoids	[57]	★★★
							Caffeic acid esters	[42]	★★
*Coleus*	*C. aliciae*	-	-	-	-	-	Diterpenoids	[58]	#
	*C. amboinicus*	Carvacrol *p*-Cymene γ-Terpinene	2 ^b^	12	[59]	★★	Phenolics	[60]	★★
*Coleus* (Cont.)	*C. amboinicus* (Cont.)	Carvacrol Caryophyllene α-Bergamotene	1 ^b^	9	[61]	★★	Terpenoids	[31]	★★★
		(E)-Caryophyllene Germacrene-D Zingiberene	1 ^a^	8	[62]	★★	Flavonoids	[63]	★★
		Carvacrol Caryophyllene α-Bergamotene	8 ^a^	32	[64]	★★	Flavonoids	[65]	★
		Carvacrol Caryophyllene β-Cymene γ-Terpinene	1 ^a^	27	[66]	★★	-	-	-
		Carvacrol β-Caryophyllene γ-Terpinene	1 ^a^	43	[67]	★★★			
		Carvacrol	1 ^a^	13	[68]	★★			
	*C. barbatus* *	Caryophyllene β-Phellandrene β-Linalool	1 ^a,b^	137	[69]	★★★	Diterpenoids	[58]	#
							Diterpenoids	[70]	#
							Phenolics	[71]	★★
							Diterpenoids	[72]	★★
							Diterpenoids	[73]	★★
							Diterpenoids	[74]	★★
							Diterpenoids	[75]	★★
							Diterpenoids	[76]	★★
							Diterpenoids	[77]	★★
*Coleus* (Cont.)	*C. caninus*	β-Caryophyllene β-Pinene Terpinyl acetate	2 ^a,b^	131	[69]	★★★	Phenylpropanoids Terpenoids	[78]	★★★
							Caffeic acid esters	[79]	★★
							Caffeic acid esters	[42]	★★
	*C. comosus* *	α-Pinene Sabinene β-Pinene	3 ^b,d^	77	[80]	★★★	Diterpenoids	[81]	★★★
		α-Thujene β-Caryophyllene	11 ^b^	33	[82]	★★	Carboxylic acid	[83]	★★★
		-	-	-	-	-	Diterpenoids	[84]	★★★
							Phenolic acids	[85]	★★
							Diterpenoids	[86]	★★★
							Diterpenoids	[87]	★★★
	*C. cylindraceus*	α-Thujene β-Maaliene	1 ^a^	23	[88]	★★	Triterpene ester Steroids	[89]	★★
							Flavonoids	[90]	★★
	*C. grandidentatus*	Camphor τ-Cadinol	1 ^a^	62	[91]	★★★	Diterpenoids	[32]	★★
							Diterpenoids	[92]	★★★
							Diterpenoids	[87]	★★★
	*C. hadiensis*	Methyl eugenol	1 ^a^	22	[93]	★★	Terpenoids	[94]	★★★
		Pipertone oxide	1 ^a^	25	[95]	★★	Diterpenoids	[96]	★★
		-	-	-	-	-	Diterpenoids	[97]	★★
							Coumarins Flavonoids Tannins Terpenes Sterols	[93]	★
							Flavonoids	[90]	★★
*Coleus* (Cont.)	*C. hereroensis*	-	-	-	-	-	Diterpenoids	[98]	★★
							Diterpenoids	[99]	★★
							Aristolane sesquiterpene aldehyde	[100]	★★
	*C. madagascariensis*	α-Fenchyl acetate β-Caryophyllene	3 ^d^	23	[101]	★★	Diterpenoids Phenolic acid	[102]	★★
							Diterpenoids Phenolics	[39]	★★★
							Diterpenoids Phenolic acid	[103]	★★
	*C. neochilus*	Caryophyllene oxide β-Caryophyllene α-Pinene	1 ^a^	31	[104]	★★	Flavonoid glycosides Polyphenols	[39]	★★★
		Aromadendrene Selina-3, 7(11)-diene	1 ^a^	80	[105]	★★	Phenolic acids	[106]	#
		β-Caryophyllene α-Pinene α-Thujene	1 ^a^	17	[107]	★★	-	-	-
	*C. porcatus*	-	-	-	-	-	Diterpenoids Phenolic acid	[108]	★★
							Diterpenoids	[109]	★★★
	*C. rotundifolius*	-	-	-	-	-	Alcohols Aldehydes Alkanes Alkyne Amines Aromatics Carboxylic acid Chloro compounds	[110]	★★★
*Coleus* (Cont.)	*C. rotundifolius* (Cont.)	-	-	-	-	-	Isocynate Isocyanides Ketones Phenols Primary alcohols Tertiary alcohols	[110]	★★★
	*C. venteri*	-	-	-	-	-	Acetophenones	[111]	★★
*Endostemon*	*E. obtusifolius*	-	-	-	-	-	Caffeic acid esters	[42]	★★
*Equilabium*	*E. petiolare*	-	-	-	-	-	Flavonoids	[90]	★★
*Hoslundia*	*H. opposita*	Eugenol	5 ^d^	88	[112]	★★★	Abietane-type esters	[113]	★★★
							Flavonoids	[114]	★★
		Camphor 1,8-Cineole	1 ^a^	37	[115]	★★	Flavonoids	[116]	★★
							Flavonoids	[117]	★★
*Lamium* *	*L. amplexicaule* *	-	1 ^a^	33	[118]	★★	Iridoid glucosides	[119]	#
		Camphor Germacrene-D	1 ^a^	48	[120]	★★	Iridoid glucosides	[121]	★ ★ ★
		-	-	-	-	-	Iridoid glucosides	[122]	★ ★ ★
							Terpenoids	[123]	★ ★
							Iridoid glucosides	[124]	★ ★
							Terpenoids	[125]	★ ★ ★
	*L. galeobdolon* *	-	1 ^a^	21	[126]	★★	Benzoxazinoids Benzoxazinones	[122]	★★★
							Iridoid glucosides	[40]	★★
*Leonotis*	*L. leonurus*	Bourbonene cis-β-Ocimene Germacrene-D Limonene α-Humulene β-Caryophyllene	3 ^d^	25	[127]	★★	Diterpenes	[128]	★★
		Germacrene-D Limonene β-Caryophyllene	1 ^a^	30	[129]	★★	Diterpenes	[130]	★★
		β-Caryophyllene Germacrene-D	1 ^a^	56	[131]	★★★	Diterpenes	[132]	★★★
		α-Pinene β-Caryophyllene	1 ^a^	33	[133]	★★	Alkaloids Dicarboxylic acid Diterpene esters Flavonoids Iridoid glycoside	[10]	#
		Caryophyllene Germacrene-D	1 ^a^	21	[134]	★★	Diterpene ester	[135]	★★★
							Unknown compounds	[136]	★
							Flavonoids	[137]	★★★
							Labdane diterpenoids	[138]	★
							Diterpenoid	[139]	★★★
							Flavonoids Labdane diterpenoids Phenolics	[140]	#
							Labdane diterpenoids	[141]	★★★
							Labdane diterpenoids	[142]	★
							Diterpenoids	[143]	★★★
							Labdane diterpenoid	[29]	★
*Leonotis* (Cont.)	*L. leonurus* (Cont.)	-	-	-	-	-	Alkaloids Flavonoids Phenolics	[144]	★★★
	*L. nepetifolia*	E-Ocimene	2 ^c^	31	[145]	★★	Diterpenoids	[146]	★★★
		β-Caryophyllene Germacrene-D δ-Selinene	1 ^a^	43	[147]	★★	Carotenoids Flavonoids Phenolics	[148]	★★
		Germacrene-D	-	3	[134]	★★	Diterpenoids	[149]	★★
	*L. ocymifolia*	Caryophyllene oxide	1 ^a^	68	[150]	★★★	Diterpenoids	[151]	★★
		Caryophyllene Germacrene	1 ^a^	21	[134]	★★			
		(Z)-β-Ocimene β-Caryophyllene Germacrene-D	1 ^a^	26	[129]	★★			
	*L. ocymifolia* var*. ocymifolia*	Caryophyllene Germacrene-D	1 ^a^	10	[134]	★★	-	-	
	*L. ocymifolia* var*. raineriana*	Germacrene-D	1 ^a^	27	[152]	★★	Diterpenoids	[153]	★★
	*L. ocymifolia* var*. schinzii*	Caryophyllene Germacrene-D	1 ^a^	16	[134]	★★	-	-	
*Leucas*	*L. capensis*	-	-	-	-	-	Diterpenoids	[154]	★★★
	*L. glabrata*	Isomenthone Piperitone Pulegone	1 ^a^	37	[155]	★★	-	-	-
	*L. martinicensis*	Germacrene-D	1 ^a^	39	[147]	★★	Alkaloids Flavonoids Glycosides Saponins	[156]	★
*Marrubium* *	*M. vulgare* *	γ-Eudesmol	1 ^a^	34	[157]	★★	Monoterpene acid	[158]	★★★
		γ-Eudesmol	1 ^b^	34	[159]	★★	Flavonoids	[160]	★★★
*Marrubium* * (Cont.)	*M. vulgare* * (Cont.)	-	-	-	-	-	Phenylethanoid Terpenoids	[160]	★★★
		β-Caryophyllene Germacrene-D	1 ^a^	12	[161]	★★	Diterpenoids	[162]	★★★
		β-Caryophyllene β-Bisabolene	1 ^a^	33	[163]	★★	Coumarins Flavonoids Phenolic acids Phenylpropanoid acids Phenylpropanoid esters Phenylpropanoid glycosides Terpenoids	[164]	#
*Mentha*	*M. aquatica*	Linalool Linalyl acetate α-Pinene	1 ^a^	42	[165]	★★	Phenolics	[166]	★★
		L-Menthone Pulegone	1 ^a^	18	[26]	★★	Phenolics	[167]	★★
		1,8-Cineole Piperitenone β-Caryophyllene	1 ^a^	29	[27]	★★	Phenolics	[168]	★★
		1,8-Cineole	1 ^a^	31	[169]	★★	Diterpenes	[170]	★★
		Limonene β-Caryophyllene Germacrene-D	1 ^b^	34	[171]	★★	Flavonoids	[172]	★★
		1,8-Cineole Methanofuran β-Caryophyllene	1 ^a^	29	[173]	★★	Flavonoids	[174]	★★
		-	-	-	-	-	Flavonoid glycones	[175]	★★★
							Monoterpene ketones	[176]	★★★
	*M. longifolia*	Isomenthone Pulegone	1 ^a^	36	[177]	★★	Flavonoids	[178]	★★★
*Mentha* (Cont.)	*M. longifolia* (Cont.)	Carvone Limonene	1 ^a^	23	[179]	★★	Flavonoids	[180]	★★★
		1,8-Cineole Menthone Pulegone	4 ^d^	34	[181]	★★	Flavonoids	[182]	★★★
		1,8-Cineole Isomenthone Pulegone	1 ^a^	30	[131]	★★	-	-	-
	*M. longifolia* subsp*. capensis*	Menthone Pulegone	2 ^a^	21	[183]	★★	-	-	-
		Menthone Pulegone	1 ^b^	31	[184]	★★			
	*M. longifolia* subsp. *polyadenia*	Methanofuran *cis*-Piperitone oxide Piperitone oxide	8 ^d^	59	[185]	★★★	-	-	-
		Methanofuran *cis*-Piperitone oxide Piperitone oxide	8 ^d^	52	[186]	★★★			
	*M. pulegium* *	Piperitone Piperitenone	1 ^a^	16	[187]	★★	Phenolics	[188]	★★★
		Menthone Pulegone	1 ^a^	53	[189]	★★★	Phenolics	[190]	★★★
*Micromeria*	*M. biflora*	Geranial Neral	2 ^c^	55	[191]	★★★	Caffeic acid	[192]	★★★
		Germacrene-D Linalool	1 ^a^	40	[193]	★★	-	-	-
*Ocimum*	*O. x africanum*	-	1 ^a^	19	[194]	★★★	-	-	-
*Ocimum* (Cont.)	*O. americanum*	-	1 ^a^	27	[194]	★★	Neolignan	[195]	★★★
		1,8-Cineole Camphor	3 ^d^	32	[196]	★★	Sesquiterpene alcohols	[197]	★★★
		1,8-Cineole Terpinen-4-ol	2 ^d^	36	[198]	★★	Flavones	[199]	★★★
		Eugenol Methyl carvacrol Terpineol	4 ^b,c^	17	[200]	★★	-	-	-
		*cis*-β-Ocimene Estragol β-Bisabolene	1 ^a^	22	[201]	★★			
		Camphor	1 ^c^	51	[202]	★★★			
		Carvone Elemol α-Humulene	1 ^a^	41	[131]	★★			
		1,8-Cineole (Z)-Methyl cinnamate	1 ^a^	14	[203]	★★			
	*O. gratissimum*	Eugenol Germacrene-D	1 ^c^	35	[202]	★★	Caffeic acid esters	[42]	★
	*O. labiatum*	-	-	-	-	-	Diterpenoids	[204]	★★
							Terpenoids	[205]	★★★
	*O. obovatum*	-	1 ^a^	43	[206]	★★	Terpenoid saponins	[207]	★★★
*Orthosiphon*	*O. thymiflorus*	2-isopropyl-5-methyl-9-methylene-bicyclo-1-decene(4.4.0)	1 ^a^	33	[208]	★★	-	-	-
*Platostoma*	*P. rotundifolium*	Germacrene-D β-Caryophyllene β-Gurjunene	1 ^d^	24	[209]	★★	Terpenoids	[210]	#
		Spathulenol	1 ^a^	59	[211]	★★★			
*Plectranthus*	*P. ambiguus*	-	-	-	-	-	Terpenoids	[58]	#
							Caffeic acid esters	[42]	★
							Flavonoids	[90]	★★★
							Phyllocladanes	[212]	★★★
	*P. cilliatus*	Bicyclogermacrene Spathulenol	2 ^d^	61	[91]	★★★	Terpenoids	[58]	#
		Spathulenol Bicyclogermacrene δ-Cadinine	2 ^d^	106	[213]	★★★			
	*P. ecklonii*	-	-	-	-	-	Terpenoids	[58]	#
							Flavonoids	[90]	★★★
	*P. ernestii*	-	-	-	-	-	Terpenoids	[212]	#
							Terpenoids	[214]	★★★
	*P. fruticosus*	Sabinyl acetate	1 ^c^	52	[215]	★★★	Terpenoids	[216]	★★★
							Terpenoids	[210]	#
	*P. lucidus*	-	-	-	-	-	Terpenoids	[96]	★★★
							Terpenoids	[97]	★★
	*P. praetermissus*	-	-	-	-	-	Terpenoids	[96]	★★★
	*P. purpuratus*	-	-	-	-	-	Diterpenoid quinomethans Vinylogous quinones Phyllocladene derivative	[217]	★★★
	*P. purpuratus* subsp*. purpuratus*	-	-	-	-	-	Terpenoids	[97]	★★
*Plectranthus* (Cont.)	*P. saccatus*	-	-	-	-	-	Terpenoids	[218]	★★
							Terpenoids	[219]	★★★
	*P. strigosus*	-	-	-	-	-	Terpenoids	[58]	#
							Terpenoids	[210]	#
							Terpenoids	[220]	#
	*P. verticillatus*	-	-	-	-	-	Terpenoids	[221]	★★
							Terpenoids	[222]	★★★
	*P. zuluensis*	-	-	-	-	-	Terpenoids	[221]	★★
							Terpenoids	[96]	★★★
							Terpenoids	[213]	★★★
							Terpenoids	[219]	★★★
*Prunella* *	*P. vulgaris* *	Germacrene-D	1 ^a^	28	[223]	★★	Pentacyclic triterpenoid Flavonoids Flavonoid glycosides Phytosterols	[224]	★★
		Aromadendrene	1 ^c^	28	[225]	★★	Terpenoids	[226]	★★★
		-	-	-	-	-	Terpenoids	[227]	★★★
							Terpenoids	[228]	★★★
		-	-	-	-	-	Triterpenoids Saponins Sterols Flavonoids Coumarins Phenylpropanoids	[229]	#
*Pseudodictamnus*	*P. africanus*	-	-	-	-	-	Terpenes	[230]	★★★
							Terpenes Phenolics	[231]	★★★
							Terpenes	[232]	★★★
*Rotheca*	*R. myricoides*	-	-	-	-	-	Cyclohexapeptide	[233]	★★★
	*R. wildii*	-	-	-	-	-	Saponins	[234]	#
							Triterpenoid Saponins	[235]	#
*Salvia*	*S. africana*	*p*-Cymene α-Eudesmol γ-Terpinene	1 ^b^	53	[236]	★★★	Diterpenoids	[237]	★★
		Caryophyllene oxide β-Caryophyllene	1 ^a^	56	[238]	★★★	Terpenoids	[204]	★★
		Caryophyllene oxide Spathulenol	1 ^a^	45	[239]	★★	Phenolics	[240]	★★★
		Limonene Viridiflorol	12 ^d^	46	[241]	★★			
	*S. albicaulis*	Viridiflorol	1 ^a^	38	[242]	★★	Phenolics	[240]	★★★
	*S. aurea*	Caryophyllene oxide α-Eudesmol β-Eudesmol	1 ^a^	48	[238]	★★	Terpenoids	[243]	★★
		Myrcene	1 ^a^	43	[239]	★★	Phenolics	[244]	★★
		Terpinene-4-ol + β-Caryophyllene α-Eudesmol β-Eudesmol	6 ^d^	20	[245]	★★	Phenolics	[240]	★★★
		Limonene α-Humulene β-Caryophyllene + Terpinen-4-ol τ-Cadinol	1 ^a^	43	[131]	★★	-	-	-
	*S. aurita*	-	-	-	-	-	Phenolics	[240]	★★★
*Salvia* (Cont.)	*S. chamelaeagnea*	1,8-Cineole α-Pinene	1 ^a^	43	[239]	★★	Terpenoids	[210]	#
		Viridiflorol	5 ^d^	18	[245]	★★	Phenolics	[240]	★★★
							Terpenoids	[246]	★★★
	*S. coccinea*	2,5-Dimethoxy-*p*-cymene Acenaphthene Aromadendrene Globulol	3 ^c^	21	[247]	★★★	Phenolics	[248]	★★
	*S. dentata*	-	-	-	-	-	Phenolic diterpene	[249]	★
	*S. disermas*	Linalyl acetate Shyobunone	3 ^d^	28	[238]	★★	Phenolic diterpene	[249]	★
		Linalool Linalyl acetate	1 ^a^	42	[131]	★★	Phenolics	[240]	★★★
	*S. dolomitica*	1,8-Cineole Borneol β-Caryophyllene	3 ^b,d^	110	[250]	★★★	Phenolics	[240]	★★★
		β-Caryophyllene Limonene Germacrene-D	3 ^b^	37	[251]	★★			
		1,8-Cineole β-Caryophyllene	1 ^a,b^	46	[252]	★★			
		1,8-Cineole β-Caryophyllene	12 ^d^	66	[238]	★★★			
		Linalool	1 ^a^	34	[242]	★★	-	-	-
	*S. garipensis*	-	-	-	-	-	Phenolics	[240]	★★★
	*S. lanceolata*	Sabinene Spathulenol	1 ^a^	41	[238]	★★	Phenolic diterpene	[249]	★
*Salvia* (Cont.)	*S. lanceolata* (Cont.)	Caryophyllene oxide Spathulenol	1 ^a^	43	[239]	★★	Phenolics	[240]	★★★
		β-Caryophyllene	12 ^d^	48	[241]	★★	-	-	-
		Bicyclogermacrene Terpinene-4-ol + β-Caryophyllene	5 ^d^	15	[245]	★★			
	*S. muirii*	1,8-Cineole Limonene α-Pinene	1 ^a^	39	[253]	★★	Phenolics	[240]	★★★
	*S. namaensis*	Camphene Camphor	1 ^a^	55	[238]	★★★	Phenolic diterpene	[249]	★
		1,8-Cineole Camphene Camphor α-Pinene	1 ^a^	20	[254]	★★	Phenolics	[240]	★★★
		Camphor	1 ^a^	64	[131]	★★★			
	*S. radula*	-	-	-	-	-	Phenolics	[240]	★★★
	*S. repens*	Ledol α-Bisabolol β-Phellandrene δ-3-Carene E-Nerolidol	6 ^d^	106	[255]	★★★	Phenolic diterpene	[249]	★
		β-Phellandrene β-Caryophyllene	1 ^a^	55	[256]	★★★	Terpenoids	[257]	★★★
		Ledol α-Bisabolol β-Caryophyllene β-Phellandrene E-Nerolidol	4 ^d^	90	[258]	★★★	Terpenoids	[259]	★★★
							Phenolics	[240]	★★★
*Salvia* (Cont.)	*S. runcinata*	Caryophyllene Ledol β-Bisabolone	2 ^b^	26	[260]	★★	Phenolic diterpene	[249]	★
		α-Bisabolol	1 ^a^	24	[238]	★★	Phenolics	[240]	★★★
		α-Pinene β-Caryophyllene E-Nerolidol	15 ^d^	157	[255]	★★★			
		α-Bisabolol β-Caryophyllene	1 ^a^	73	[256]	★★★			
		Limonene Nerolidol α-Bisabolol β-Caryophyllene β-Eudesmol δ-3-Carene	20 ^d^	11 (x > 5%)	[261]	★★			
		Viridiflorol β-Caryophyllene	1 ^a^	44	[131]	★★			
		α-Bisabolol β-Caryophyllene E-Nerolidol	12 ^d^	118	[258]	★★★			
	*S. scabra*	-	-	-	-	-	Phenolic diterpene	[249]	★
	*S. schlechteri*	-	-	-	-	-	Phenolics	[240]	★★★
	*S. stenophylla*	Myrcene α-Bisabolol	1 ^a^	34	[238]	★★	Phenolic diterpene	[249]	★
		α-Bisabolol δ-3-Carene	10 ^d^	128	[255]	★★★	Flavonoids	[262]	★★
		(+)-α-Bisabolol α-Phellandrene	1 ^a^	31	[263]	★★	Phenolics	[240]	★★★
		δ-3-Carene Manool	1 ^a^	59	[256]	★★★			
*Salvia* (Cont.)	*S. stenophylla* (Cont.)	δ-3-Carene Mycrene Limonene β-Phellandrene γ-Terpinene *p*-Cymene Nerolidiol α-Bisabolol	27 ^d^	12 (x > 5%)	[261]	★★	-	-	-
		δ-3-Carene *p*-Cymene (E)-nerolidiol α-Bisabolol	12 ^d^	128	[258]	★★★			
	*S. verbenaca* *	-	1 ^a^	76	[264]	★★★	Terpenoids	[210]	#
		-	2 ^a,b^	63	[265]	★★★	Phenolics	[266]	★★
		-	1 ^a^	18	[267]	★★	-	-	-
*Satureja* *	*S. thymbra* *	*p*-Cymene γ-Terpinene Thymol Carvacrol	7 ^c^	40	[268]	★★	Phenolics	[269]	★★
*Stachys*	*S. aethiopica*	-	-	-	-	-	Saponins Tannins	[270]	★★
*Syncolostemon*	*S. argenteus*	-	-	-	-	-	Pyrones	[271]	★★★
	*S. bracteosus*	-	-	-	-	-	Alcohols Acids Esters Lactones Phenolics Sesquiterpenes	[272]	★★
*Syncolostemon* (Cont.)	*S. densiflorus*	-	-	-	-	-	Pyrones	[37]	★★★
							Pyrones	[273]	★★★
	*S. modestus*	*p*-Cymene Spathulenol Viridiflorol γ-Terpinene	1 ^a^	10	[131]	★★	-	-	-
	*S. parviflorus*	-	-	-	-	-	Pyrones	[231]	★★★
	*S. petiolatus*	(+)-Linalool (E)-β-farnesene (+)-Bicyclogermacrene (−)-Germacrene D	1 ^a^	N/A	[274]	★★	-	-	-
	*S. pretoriae*	β-Caryophyllene	1 ^a^	6	[131]	★★	-	-	-
	*S. rotundifolius*	-	-	-	-	-	Pyrones	[231]	★★★
*Tetradenia*	*T. barberae*	-	-	-	-	-	Pyrones	[275]	★★★
	*T. riparia*	14-Hydroxy-9-epi-(E)-Caryophyllene 6,7-Dehydroroyleanone α-Cadinol	2 ^c^	51	[276]	★★★	Pyrones	[35]	★★★
		Fenchone δ-Cadinene	1 ^a^	49	[277]	★★	Pyrones	[278]	★★★
		14-Hydroxy-9-epi-β-Caryophyllene Fenchone	1 ^a^	64	[278]	★★★	Pyrones	[279]	★★★
		Fenchone	2 ^b^	27	[280]	★★	Pyrones	[281]	★★★
		-	-	-	-	-	Pyrones	[282]	★★★
							Terpenoids	[283]	★★★
*Tetradenia* (Cont.)	*T. riparia* (Cont.)	-	-	-	-	-	Terpenoids	[284]	★★★
							Terpenoids	[285]	★★★
							Terpenoids	[286]	★★★
							Terpenoids	[287]	★★★
							Terpenoids	[288]	★★★
*Teucrium*	*T. africanum*	α-Cubebene β-Cubebene	3 ^d^	29	[289]	★★	Terpenoids	[33]	★★★
							Terpenoids	[210]	#
	*T. sessiliflorum*	α-Cubebene β-Cubebene	3 ^d^	29	[289]	★★	Terpenoids	[290]	★★★
*Vitex*	*V. ferruginea*	Germacrene-D	2 ^d^	53	[291]	-	-	-	-
	*V. obovata*	1,8-Cineole α-Copaene	1 ^a^	92	[292]	★★★	Terpenoids	[210]	#
	*V. pooara*	Limonene Cryptone β-Selinene	1 ^a^	61	[292]	★★★	Terpenoids	[210]	#
	*V. rehmannii*	Caryophyllene oxide Spathulenol	1 ^a^	60	[292]	★★★	Terpenoids	[210]	#
	*V. trifolia* *	α-Pinene 1,8-Cineole Terpinyl acetate	1 ^a^	30	[293]	★★	Flavones	[294]	★★
							Terpenoids	[295]	★★★
	*V. zeyheri*	1,8-Cineole	1 ^a^	58	[292]	★★★	Terpenoids	[210]	#
*Volkameria*	*V. glabra*	-	-	-	-	-	Terpenoids	[210]	#
							Terpenoids	[296]	★★★
							Terpenoids	[297]	★★
							Terpenoids	[298]	★★

^a^ Samples collected from one locality with no mention of replicates, ^b^ Cultivated/grown from seed/propagated, ^c^ Seasonal samples/different vegetative stages, ^d^ More than one locality specified, # Review article data, ^ Compounds occurring greater than 10% are listed as major compounds, * Naturalized exotic species. Essential oil studies: ★ = GC Trace peaks, no identity, ★★ = <10 compounds identified, ★★★ = many compounds identified. Phytochemical studies: ★ = older studies that are not very informative, TLC or similar used, ★★ = phytochemical screening and color tests, and ★★★ = studies using modern chemical methods such as mass spectrometry and nuclear magnetic resonance spectroscopy for identification and/ or structural elucidation. Due to space limitations, author citations have been listed in Table S1.

## Data Availability

The geographical data presented in this study are openly available in GBIF (Global Biodiversity Information Facility) at https://doi.org/10.15468/dl.b8zqjh (accessed on 16 May 2021).

## Supplementary Materials

The following are available online, Table S1: List of all southern African Lamiaceae (naturalized non-indigenous species are indicated with an asterisk), Table S2. Publications consulted for ethnobotanical data.
