# Supplementary material for: The Botanical, Chemical and Ethnobotanical Diversity of Southern African Lamiaceae
Source: Molecules. 2021 Jun 18;26(12):3712. doi: 10.3390/molecules26123712 (PMC8233991; doi:10.3390/molecules26123712)
Supplement: Supplementary file 1 [file molecules-26-03712-s001.zip › molecules-1248762-supplementary.pdf]

molecules

MDPI

Table S1. List of all southern African Lamiaceae (naturalized non-indigenous species are indicated with an asterisk).

Genus	Species
Acrotome Benth. ex Endl.	*Acrotome angustifolia* G.Taylor.
	Acrotome fleckii (Gürke) Launert
	Acrotome hispida Benth.
	Acrotome inflata Benth.
	Acrotome pallescens Benth.
	Acrotome thorncroftii Skan
Aeollanthus C.Mart. ex Spreng.	*Aeollanthus buchnerianus* Briq.
	Aeollanthus namibiensis Ryding
	Aeollanthus neglectus (Dinter) Launert
	Aeollanthus parvifolius Benth.
	Aeollanthus rehmannii Gürke
	Aeollanthus suaveolens Mart. ex Spreng.
Ajuga L.	*Ajuga ophrydis* Burch. ex Benth
Basilicum Moench	*Basilicum polystachyon* (L.) Moench
Cantinoa Harley & J.F.B.Pastore*	*Cantinoa americana* (Aubl.) Harley & J.F.B.Pastore [=*Hyptis spicigera* Lam.]*
	Cantinoa mutabilis (Rich.) Harley & J.F.B.Pastore [=*Hyptis mutabilis* (Rich.) Briq.]*
Cedronella Moench*	*Cedronella canariensis* (L.) Webb & Berthel.*
Clerodendrum L.	*Clerodendrum africanum* Moldenke
	Clerodendrum buchneri Gürke
	Clerodendrum bungei Steud.*
	Clerodendrum pleiosciadium Gürke
	Clerodendrum ternatum Schinz [=*C. transvaalense* B.Thomas]
Coleus Lour.	*Coleus aliciae* (Codd) A.J.Paton [=*Plectranthus aliciae* (Codd) van Jaarsv. & T.J.Edwards]
	Coleus amboinicus Lour. [=*Plectranthus amboinicus* (Lour.) Spreng.]
	Coleus barbatus (Andrews) Benth. ex G.Don [=*Plectranthus barbatus* Andrews]*
	Coleus barbatus var. grandis (L.H.Cramer) A.J.Paton [=*Plectranthus barbatus var. grandis* (L.H.Cramer) Lukhoba & A.J.Paton]*
	Coleus bojeri Benth. [=*Solenostemon latifolius* (Hochst. ex Benth.) J.K.Morton]
	Coleus calycinus (Benth.) A.J.Paton [=*Isodon calycinus* (Benth.) H.W.Li and *Rabdosiella calycina* (Benth.) Codd]
	Coleus caninus (Roth) Vatke [=*Plectranthus caninus* Roth]
	Coleus comosus Hochst. ex Gürke [=*Plectranthus ornatus* Codd]*
	Coleus cylindraceus (Hochst. ex Benth.) A.J.Paton [=*Plectranthus montanus* Benth.]
	Coleus dinteri (Briq.) A.J.Paton [=*Plectranthus dinteri* Briq.]
	Coleus dolichopodus (Briq.) A.J.Paton [=*Plectranthus dolichopodus* Briq.]

Genus	Species
	Coleus esculentus (N.E.Br.) G.Taylor [=*Plectranthus esculentus* N.E.Br.]
	Coleus gibbosus A.J.Paton [=*Plectranthus rehmannii* Gürke]
	Coleus gracilipedicellatum (Robyns & Lebrun) A.J.Paton [=*Holostylon baumii* (Gürke) G.Taylor]
	Coleus grandidentatus (Gürke) A.J.Paton [=*Plectranthus grandidentatus* Gürke]
	Coleus guerkei (Briq.) A.J.Paton [=*Neohyptis paniculata* (Baker) J.K.Morton]
	Coleus hadiensis (Forssk.) A.J.Paton [=*P. cyaneus* Gürke, *P. hadiensis* (Forssk.) Schweinf. ex Sprenger, *P. hadiensis* (Forssk.) Schweinf. ex Spreng. *var. hadiensis*, *P. hadiensis* (Forssk.) Schweinf. ex Spreng. *var. tomentosus* (Benth.) Codd, *P. hadiensis* (Forssk.) Schweinf. ex *Spreng. var. woodii* (Gürke) Codd and *P.ramosior* (Benth.) van Jaarsv.]
	Coleus hereroensis (Engl.) A.J.Paton [=*Plectranthus hereroensis* Engl.]
	Coleus kirkii (Baker) A.J.Paton [=*Pycnostachys reticulata* (E.Mey.) Benth.]
	Coleus lasianthus Gürke [=*Plectranthus tetensis* (Baker) Agnew]
	Coleus livingstonei A.J.Paton [=*Pycnostachys urticifolia* Hook.]
	Coleus madagascariensis (Pers.) A.Chev. [=*Plectranthus madagascariensis* (Pers.) Benth. and *Plectranthus madagascariensis* (Pers.) Benth. *var. madagascariensis*]
	Coleus mutabilis (Codd) A.J.Paton [=*Plectranthus mutabilis* Codd]
	Coleus neochilus (Schltr.) Codd [=*Plectranthus neochilus* Schltr]
	Coleus pentheri Gürke [=*Plectranthus pentheri* (Gürke ex Zahlbr.) van Jaarsv. & T.J.Edwards]
	Coleus porcatus (van Jaarsv. & P.J.D.Winter) A.J.Paton [=*Plectranthus porcatus* van Jaarsv. & P.J.D.Winter]
	Coleus psammophilus (Codd) A.J.Paton [=*Plectranthus psammophilus* Codd]
	Coleus rhodesianum (N.E.Br.) A.J.Paton [=*Englerastrum schweinfurthii* Briq.]
	Coleus rotundifolius (Poir.) A.Chev. & Perrot [=*Plectranthus rotundifolius* (Poir.) Spreng. and *Solenostemon rotundifolius* (Poir.) J.K.Morton]
	Coleus stenostachys (Baker) A.J.Paton & Phillipson [=*Pycnostachys coerulea* Hook.]
	Coleus subspicatus (Hochst.) Walp. [=*Plectranthus spicatus* E. Mey. ex Benth.]
	Coleus tetragonus (Gürke) Robyns & Lebrun [=*Plectranthus tetragonus* Gürke]
	Coleus unguentarius (Codd) A.J.Paton [=*Plectranthus unguentarius* Codd]
	Coleus venteri (van Jaarsv. & L.Hankey) A.J.Paton [=*Plectranthus venteri* van Jaarsv. & L.Hankey]
	Coleus xerophilus (Codd) A.J.Paton [=*Plectranthus xerophilus* Codd]
Endostemon N.E.Br.	*Endostemon obtusifolius* (E.Mey.) N.E.Br.
	Endostemon tenuiflorus (Benth.) M.R.Ashby
	Endostemon tereticaulis (Poir.) M.R.Ashby
Equilabium Mwany., A.J.Paton & Culham	*Equilabium candelabriforme* (Launert) Mwany. & A.J.Paton [=P*lectranthus candelabriformis* Launert]

Genus	Species
	Equilabium dolomiticum (Codd) Mwany. & A.J.Paton [=*Plectranthus dolomiticus* Codd]
	Equilabium laxiflorum (Benth.) Mwany. & A.J.Paton [=*Plectranthus laxiflorus* Benth.]
	Equilabium petiolare (Benth.) Mwany. & A.J.Paton [=*Plectranthus petiolaris* E. Mey. ex Benth.]
Haumaniastrum P.A.Duvign. & Plancke	*Haumaniastrum sericeum* (Briq.) A.J.Paton [=*Acrocephalus sericeus* Briq.]
Hoslundia Vahl	*Hoslundia opposita* Vahl
Khalarahia Baill	*Kalaharia uncinata* (Schinz) Moldenke [=*Rotheca uncinata* (Schinz) P.P.J.Herman & Retief]
Karomia Dop	*Karomia speciosa* (Hutch. & Corbishley) R.Fern. [=*K. speciosa* (Hutch. & Corbishley) R.Fern. *forma flava* (Moldenke) R.Fern.]
	Karomia speciosa (Hutch. & Corbishley) R.Fern. *forma speciosa*
Killickia Bräuchler, Heubl Doroszenko	*Killickia compacta* (Killick) Bräuchler, Heubl & Doroszenko
	Killickia grandiflora (Killick) Bräuchler, Heubl & Doroszenko
	Killickia lutea Bräuchler
	Killickia pilosa (Benth.) Bräuchler, Heubl & Doroszenko
Lamium L.*	*Lamium amplexicaule* L.*
	Lamium galeobdolon (L.) L.*
Leonotis (Pers.) R.Br.	*Leonotis leonurus* (L.) R.Br.
	Leonotis nepetifolia (L.) R.Br.
	Leonotis ocymifolia (Burm.f.) Iwarsson [=*L. dubia* E.Mey. ex Benth.]
	Leonotis ocymifolia (Burm.f.) Iwarsson *var. ocymifolia* [=*Leonotis leonitis* (L.) R.Br. and *Leonotis mollis* Benth.]
	Leonotis ocymifolia var. raineriana (Vis.) Iwarsson [=*L. intermedia* Lindl.]
	Leonotis ocymifolia var. schinzii (Gürke) Iwarsson [=*L. randii* S.Moore and *L. schinzii* Gürke]
Leucas R.Br.	*Leucas capensis* (Benth.) Engl. [=*Leonotis pentadentata* J.C.Manning Goldblatt]
	Leucas ebracteata var. kaokoveldensis Sebald [=*Leonotis ebracteata var. kaokoveldensis* (Sebald) J.C.Manning & Goldblatt]
	Leucas glabrata (Vahl) Sm. [=*Leonotis glabrata* (Vahl) J.C.Manning & Goldblatt *var. glabrata* and *Leonotis glabrata var. linearis* (Codd) J.C.Manning & Goldblatt]
	Leucas lavandulifolia Sm.*
	Leucas martinicensis (Jacq.) R.Br. [=*Leonotis martinicensis* (Jacq.) J.C.Manning Goldblatt]
	Leucas neuflizeana Courbon [=*Leonotis neuflizeana* (Courbon) J.C.Manning & Goldblatt]
	Leucas pechuelii (Kuntze) Baker [=*Leonotis pechuelii* (Kuntze) J.C.Manning & Goldblatt]
	Leucas sexdentata Skan [=*Leonotis sexdentata* (Skan) J.C.Manning & Goldblatt]
Marrubium L.*	*Marrubium vulgare* L.*

Genus	Species
Mentha L.	*Mentha aquatica* L.
	Mentha longifolia (L.) L.
	Mentha longifolia subsp. capensis (Thunb.) Briq
	Mentha longifolia subsp. polyadena (Briq.) Briq.
	Mentha longifolia subsp. wissii (Launert) Codd
	Mentha pulegium L.*
Mesosphaerum P.Browne*	*Mesosphaerum pectinatum* (L.) Kuntze*
Micromeria Benth.	*Micromeria biflora* (Buch.-Ham. ex D.Don) Benth. [=*Satureja biflora* (Buch.-Ham. ex D.Don) Briq.]
Ocimum L.	*Ocimum africanum* Lour.
	Ocimum americanum L.
	Ocimum americanum L. *var. americanum*
	Ocimum angustifolium Benth.
	Ocimum burchellianum Benth.
	Ocimum coddii (S.D.Williams & K.Balkwill) A.J.Paton
	Ocimum dolomiticola A.J.Paton
	Ocimum filamentosum Forssk. [=*Becium filamentosum* (Forssk.) Chiov.]
	Ocimum gratissimum L. [=*Ocimum urticifolium* Roth]
	Ocimum gratissimum L. *subsp. gratissimum var. gratissimum*
	Ocimum labiatum (N.E.Br.) A.J.Paton
	Ocimum natalense Ayob. ex A.J.Paton
	Ocimum obovatum E.Mey. ex Benth. [=*Becium grandiflorum var. obovatum* (E.Mey. ex Benth.) Sebald]
	O. obovatum E.Mey. ex Benth. *subsp. obovatum var. obovatum*
	Ocimum obovatum E.Mey. ex Benth. *subsp. obovatum var. galpinii* (Gürke) A.J.Paton
	Ocimum pseudoserratum (M.R.Ashby) A.J.Paton
	Ocimum reclinatum (S.D.Williams & M.Balkwill) A.J.Paton
	Ocimum serratum (Schltr.) A.J.Paton
	Ocimum tubiforme (R.D.Good) A.J.Paton
	Ocimum waterbergense (S.D.Williams & K.Balkwill) A.J.Paton
Orthosiphon Benth	*Orthosiphon fruticosus* Codd
	Orthosiphon rubicundus (D.Don) Benth.
	Orthosiphon thymiflorus (Roth) Sleesen [=*O.marmoritis* (Hance) Dunn and *O. suffrutescens* (Schumach.) J.K.Morton]
	Orthosiphon vernalis Codd
Platostoma P.Beauv.	*Platostoma rotundifolium* (Briq.) A.J.Paton [=*Geniosporum rotundifolium* Briq.]
Plectranthus L'Hér	*Plectranthus ambiguus* (Bolus) Codd
	Plectranthus brevimentum T.J.Edwards
	Plectranthus ciliatus E.Mey.

Genus	Species
	Plectranthus ecklonii Benth.
	Plectranthus elegantulus Briq.
	Plectranthus ernstii Codd
	Plectranthus fruticosus L'Hér.
	Plectranthus grallatus Briq.
	Plectranthus hilliardiae Codd
	Plectranthus hilliardiae Codd *subsp. hilliardiae*
	Plectranthus hilliardiae subsp. australis van Jaarsv. & A.E.van Wyk
	Plectranthus lasianthus (Gürke) Vollesen
	Plectranthus lucidus (Benth.) van Jaarsv. & T.J.Edwards
	Plectranthus malvinus van Jaarsv. & T.J.Edwards
	Plectranthus mirabilis (Briq.) Launert
	Plectranthus mzimvubuensis van Jaarsv.
	Plectranthus oertendahlii Th. Fr. Jr.
	Plectranthus oribiensis Codd
	Plectranthus praetermissus Codd
	Plectranthus purpuratus Harv.
	Plectranthus purpuratus Harv. *subsp. purpuratus*
	Plectranthus purpuratus subsp. montanus van Jaarsv. & T.J.Edwards
	Plectranthus purpuratus subsp. tongaensis van Jaarsv. & T.J.Edwards
	Plectranthus reflexus van Jaarsv. & T.J.Edwards
	Plectranthus rubropunctatus Codd
	Plectranthus saccatus Benth.
	Plectranthus saccatus subsp. pondoensis van Jaarsv. & T.J.Edwards
	Plectranthus saccatus var. longitubus Codd
	Plectranthus saccatus var. saccatus Benth.
	Plectranthus strigosus Benth.
	Plectranthus stylesii T.J.Edwards
	Plectranthus spicatus E.Mey.
	Plectranthus swynertonii S.Moore
	Plectranthus verticillatus (L.f.) Druce
	Plectranthus zuluensis T.Cooke
Premna L.	*Premna senensis* Klotzsch
Prunella L.*	*Prunella vulgaris* L. [=*P. vulgaris var. vulgaris*]*
Pseudodictamnus Fabr.	*Pseudodictamnus africanus* (L.) Salmaki & Siadat [=*Ballota africana* (L.) Benth.]
Rabdosiella Codd.	*Rabdosiella leemannii* N.Hahn
Rotheca Raf.	*Rotheca caerulea* (N.E.Br.) P.P.J.Herman & Retief [=*Clerodendrum caeruleum* N.E.Br.]
	Rotheca cuneiformis (Moldenke) P.P.J.Herman & Retief

Genus	Species
	Rotheca hirsuta (Hochst.) R.Fern.
	Rotheca hirsuta (Hochst.) R.Fern. *forma hirsuta*
	Rotheca hirsuta (Hochst.) R.Fern. *forma triphylla* (Harv.) Fernald
	Rotheca louwalbertsii (P.P.J.Herman) P.P.J.Herman & Retief
	Rotheca makanjana (H.J.P.Winkl.) Steane & Mabb. [=*Clerodendrum makanjanum* H.J.P.Winkl.]
	Rotheca myricoides (Hochst.) Steane & Mabb.
	Rotheca myricoides (Hochst.) Steane & Mabb. *subsp. myricoides var. myricoides*
	Rotheca pilosa (H.Pearson) P.P.J.Herman & Retief
	Rotheca suffruticosa (Gürke) Verdc. [=*Clerodendrum suffruticosum* Gürke, *C. suffruticosum* Gürke *var. natalense* Moldenke and *C. suffruticosum* Gürke *var. suffruticosum*]
	Rotheca violacea (Gürke) Verdc. [=*Clerodendrum violaceum* Gürke]
	Rotheca wildii (Moldenke) R.Fern.
	Rotheca wildii (Moldenke) R.Fern. *forma glabra* (R.Fern.) R.Fern.
Salvia L.	*Salvia africana* L. [=*S. africana-caerulea* L.]
	Salvia albicaulis Benth.
	Salvia aurea L. [=*S. africana-lutea* L.]
	Salvia aurita L.f.
	Salvia aurita L.f. *var. aurita*
	Salvia aurita var. galpinii (Skan) Hedge
	Salvia chamelaeagnea Berg.
	Salvia coccinea Buc'hoz ex Etl. [=*S. coccinea* L.f.]
	Salvia dentata Aiton
	Salvia disermas L.
	Salvia dolomitica Codd
	Salvia garipensis E. Mey.
	Salvia granitica Hochst.
	Salvia lanceolata Lam.
	Salvia muirii L.Bolus
	Salvia namaensis Schinz
	Salvia obtusata Thunb.
	Salvia radula Benth.
	Salvia reflexa Hornem.*
	Salvia repens Burch. ex Benth.
	Salvia repens Burch. ex Benth. *var. repens*
	Salvia repens var. keiensis Hedge
	Salvia repens var. transvaalensis Hedge
	Salvia runcinata L.f.
	Salvia scabra Thunb.

Genus	Species
	Salvia schlechteri Briq.
	Salvia sclarea L.*
	Salvia stenophylla Burch. ex Benth.
	Salvia thermarum van Jaarsv.
	Salvia tiliifolia Vahl*
	Salvia triangularis Thunb.
	Salvia tysonii Skan
	Salvia verbenaca L.*
Satureja L.*	*Satureja thymbra* L.*
*Scutellaria**	*Scutellaria racemosa* Pers.*
Stachys L.	*Stachys aethiopica* L.
	Stachys albiflora N.E.Br.
	Stachys arachnoidea Codd
	Stachys arvensis (L.) L.*
	Stachys aurea Benth.
	Stachys bolusii Skan
	Stachys burchelliana Launert
	Stachys caffra E.Mey. ex Benth.
	Stachys comosa Codd
	Stachys cuneata Banks ex Benth.
	Stachys cymbalaria Briq.
	Stachys dinteri Launert
	Stachys dregeana Benth
	Stachys erectiuscula Gürke
	Stachys flavescens Benth.
	Stachys flexuosa Skan
	Stachys graciliflora C.Presl
	Stachys grandifolia E. Mey.
	Stachys humifusa Burch. ex Benth.
	Stachys hyssopoides Burch. ex Benth.
	Stachys kuntzei Gürke
	Stachys lamarckii Benth.
	Stachys linearis Burch. ex Benth.
	Stachys malacophylla Skan
	Stachys natalensis Hochst.
	Stachys natalensis Hochst. *var. natalensis*
	Stachys natalensis var. galpinii (Briq.) Codd
	Stachys nigricans Benth.
	Stachys obtusifolia MacOwan

Genus	Species
	Stachys rehmannii Skan
	Stachys reticulata Codd
	Stachys rivularis J.M.Wood & M.S.Evans
	Stachys rudatisii Skan
	Stachys rugosa Aiton
	Stachys scabrida Skan
	Stachys sessilifolia E.Mey.
	Stachys sessilis Gürke
	Stachys simplex Schltr.
	Stachys spathulata Burch. ex Benth.
	Stachys sublobata Skan
	Stachys thunbergii Benth.
	Stachys tubulosa MacOwan
	Stachys tysonii Skan
	Stachys zeyheri Skan
Syncolostemon E.Mey. ex Benth.	*Syncolostemon albiflorus* (N.E.Br.) D.F.Otieno
	Syncolostemon argenteus N.E.Br.
	Syncolostemon aurulentus Ngwenya
	Syncolostemon bolusii (N.E.Br.) D.F.Otieno
	Syncolostemon bracteosus (Benth.) D.F.Otieno [=*Hemizygia bracteosa* (Benth.) Briq.]
	Syncolostemon canescens (Gürke) D.F.Otieno
	Syncolostemon cinereum (Codd) D.F.Otieno & Retief
	Syncolostemon comptonii Codd
	Syncolostemon concinnus N.E.Br.
	Syncolostemon densiflorus Benth.
	Syncolostemon elliottii (Baker) D.F.Otieno
	Syncolostemon eriocephalus I.Verd.
	Syncolostemon floccosus (Launert) D.F.Otieno
	Syncolostemon foliosus (S.Moore) D.F.Otieno
	Syncolostemon gerrardii (N.E.Br.) D.F.Otieno
	Syncolostemon incanus (Codd) D.F.Otieno
	Syncolostemon latidens (N.E.Br.) Codd
	Syncolostemon linearis (Benth.) D.F.Otieno
	Syncolostemon macranthus (Gürke) Ashby
	Syncolostemon macrophyllus Gürke
	Syncolostemon modestus (Codd) D.F.Otieno
	Syncolostemon obermeyerae (M.Ashby) D.F.Otieno
	Syncolostemon parviflorus E.Mey. ex Benth.
	Syncolostemon parviflorus E.Mey. ex Benth. *var. lanceolatus* (Gürke) Codd

Genus	Species
	Syncolostemon parviflorus E.Mey. ex Benth. *var. parviflorus*
	Syncolostemon parvifolius (Codd) D.F.Otieno
	Syncolostemon persimilis (N.E.Br.) D.F.Otieno
	Syncolostemon petiolatus (Ashby) D.F.Otieno
	Syncolostemon pretoriae (Gürke) D.F.Otieno
	Syncolostemon punctatus (Codd) D.F.Otieno
	Syncolostemon ramosus (Codd) D.F.Otieno
	Syncolostemon ramulosus E.Mey. ex Benth.
	Syncolostemon rehmannii (Gürke) D.F.Otieno
	Syncolostemon rotundifolius E.Mey. ex Benth.
	Syncolostemon rugosifolius (M.Ashby) D.F.Otieno
	Syncolostemon stalmansii (A.J.Paton & K.Balkwill) D.F.Otieno
	Syncolostemon stenophyllus (Gürke) D.F.Otieno
	Syncolostemon subvelutinus (Gürke) D.F.Otieno
	Syncolostemon teucriifolius (Hochst.) D.F.Otieno
	Syncolostemon thorncroftii (N.E.Br.) D.F.Otieno
	Syncolostemon transvaalensis (Schltr.) D.F.Otieno
	Syncolostemon welwitschii (Rolfe) D.F.Otieno [=*Orthosiphon pseudornatus* R.D.Good]
Tetradenia Benth.	*Tetradenia bainesii* (N.E.Br.) Phillipson & C.F.Steyn
	Tetradenia barberae (N.E.Br.) Codd
	Tetradenia brevispicata (N.E.Br.) Codd
	Tetradenia galpinii (N.E.Br.) Phillipson & C.F.Steyn
	Tetradenia kaokoensis van Jaarsv. & A.E.van Wyk
	Tetradenia riparia (Hochst.) Codd
	Tetradenia tuberosa T.J.Edwards
Teucrium L.	*Teucrium africanum* Thunb.
	Teucrium kraussii Codd
	Teucrium sessiliflorum Benth. [=*T. trifidum* Schltdl.]
Thorncroftia N.E.Br.	*Thorncroftia greenii* Changwe & K.Balkwill
	Thorncroftia longiflora N.E.Br.
	Thorncroftia lotteri T.J.Edwards & McMurtry
	Thorncroftia media Codd
	Thorncroftia succulenta (R.A.Dyer & E.A.Bruce) Codd
	Thorncroftia thorncroftii (S.Moore) Codd
Tinnea Kotschy ex Hookf.	*Tinnea barbata* Vollesen
	Tinnea eriocalyx Welw.
	Tinnea galpinii Briq.
	Tinnea rhodesiana S.Moore

Genus	Species
Vitex L.	*Vitex angolensis* Gürke
	Vitex ferruginea Schumach. & Thonn. [=*V. amboniensis* Gurke, *V. ferruginea* Schumach. & Thonn. *subsp. amboniensis* (Gurke) Verdc. *var. amboniensis* and *V. ferruginea* Schumach. & Thonn. *subsp. amboniensis* (Gurke) Verdc. *var. amaniensis* (W.Piep.) Verdc.]
	Vitex harveyana H.Pearson
	Vitex mombassae Vatke
	Vitex mooiensis H.Pearson [=*Premna mooiensis* (H.Pearson) W.Piep.]
	Vitex obovata E.Mey. [= *V. obovata* E.Mey. *subsp. obovata* and *V. obovata* E.Mey. *subsp. wilmsii* (Gürke) Bredenk. D.J.Botha]
	Vitex patula E.A.Bruce
	Vitex pooara Corbishley
	Vitex rehmannii Gürke
	Vitex trifolia L.*
	Vitex zeyheri Sond. ex Schauer
Volkameria P.Browne	*Volkameria glabra* (E.Mey.) Mabb. & Y.W.Yuan [=*Clerodendrum glabrum* E.Mey.]

Table S2. Publications consulted for ethnobotanical data.

Genus	Publications consulted
Acrotome	[17,327-329,342,345,365,418-420]
Aeollanthus	[327]
Ajuga	[327,328,331]
Clerodendrum	[329,330]
Coleus	[17,327,331,338,340-343,359,418]
Equilabium	[331,338,342,343,359,360,418-420]
Hoslundia	[331,342,345,418-420]
Kalaharia	[329,365,418,420]
Leonotis	[327,328,330,331,338,342,343,345,351,359-361,363-365,378,383,393-395,418-421]
Leucas	[17,327,331,338,345,351,363-365,378,383,418,420,422]
Marrubium	[327,343,369,370,420]
Mentha	[15,17,327,328,331,338,342,343,351,359,361,363,369,378,393,394,396,418-421,423,424]
Micromeria	[343]
Ocimum	[17,327-329,331,338,342,343,345,359,361,365,378,379,418-420]
Plectranthus	[327-329,331,338,340,341,359,361,364,383]
Prunella	[331]
Pseudodictamnus	[15,327,343,351,359,363,378,393,394,396,418,420,421,423,424]
Rotheca	[328,331,338,385,407,418,420]
Salvia	[15,327,328,338,343,351,359,363,365,378,393-396,418,420,421,423-425]
Stachys	[17,327,328,330,331,338,343,345,351,360,363,378,393,394,396,418,420,421]
Syncolostemon	[327,329,331,338,365,418,420]
Tetradenia	[15,327,331,338,343,359,364,369,378,383,385]
Teucrium	[15,17,327,338,343,351,363,369,378,395,418,420]
Vitex	[327,338,342,385,407,418-420]
